# Patterns of Social Determinants of Health and Child Mental Health, Cognition, and Physical Health

**DOI:** 10.1001/jamapediatrics.2023.4218

**Published:** 2023-10-16

**Authors:** Yunyu Xiao, J. John Mann, Julian Chun-Chung Chow, Timothy T. Brown, Lonnie R. Snowden, Paul Siu-Fai Yip, Alexander C. Tsai, Yu Hou, Jyotishman Pathak, Fei Wang, Chang Su

**Affiliations:** 1 Department of Population Health Sciences, Weill Cornell Medicine, New York, New York; 2 Departments of Psychiatry and Radiology, Columbia University Irving Medical Center, Columbia University, New York, New York; 3 Division of Molecular Imaging and Neuropathology, New York State Psychiatric Institute, New York; 4 School of Social Welfare, University of California, Berkeley; 5 School of Public Health, University of California, Berkeley; 6 Department of Social Work and Social Administration, The University of Hong Kong, Hong Kong, China; 7 Hong Kong Jockey Club Centre for Suicide Research and Prevention, Hong Kong, China; 8 Center for Global Health and Mongan Institute, Massachusetts General Hospital, Boston; 9 Harvard Medical School, Boston, Massachusetts

## Abstract

**Importance:**

Social determinants of health (SDOH) influence child health. However, most previous studies have used individual, small-set, or cherry-picked SDOH variables without examining unbiased computed SDOH patterns from high-dimensional SDOH factors to investigate associations with child mental health, cognition, and physical health.

**Objective:**

To identify SDOH patterns and estimate their associations with children’s mental, cognitive, and physical developmental outcomes.

**Design, Setting, and Participants:**

This population-based cohort study included children aged 9 to 10 years at baseline and their caregivers enrolled in the Adolescent Brain Cognitive Development (ABCD) Study between 2016 and 2021. The ABCD Study includes 21 sites across 17 states.

**Exposures:**

Eighty-four neighborhood-level, geocoded variables spanning 7 domains of SDOH, including bias, education, physical and health infrastructure, natural environment, socioeconomic status, social context, and crime and drugs, were studied. Hierarchical agglomerative clustering was used to identify SDOH patterns.

**Main Outcomes and Measures:**

Associations of SDOH and child mental health (internalizing and externalizing behaviors) and suicidal behaviors, cognitive function (performance, reading skills), and physical health (body mass index, exercise, sleep disorder) were estimated using mixed-effects linear and logistic regression models.

**Results:**

Among 10 504 children (baseline median [SD] age, 9.9 [0.6] years; 5510 boys [52.5%] and 4994 girls [47.5%]; 229 Asian [2.2%], 1468 Black [14.0%], 2128 Hispanic [20.3%], 5565 White [53.0%], and 1108 multiracial [10.5%]), 4 SDOH patterns were identified: pattern 1, affluence (4078 children [38.8%]); pattern 2, high-stigma environment (2661 children [25.3%]); pattern 3, high socioeconomic deprivation (2653 children [25.3%]); and pattern 4, high crime and drug sales, low education, and high population density (1112 children [10.6%]). The SDOH patterns were distinctly associated with child health outcomes. Children exposed to socioeconomic deprivation (SDOH pattern 3) showed the worst health profiles, manifesting more internalizing (β = 0.75; 95% CI, 0.14-1.37) and externalizing (β = 1.43; 95% CI, 0.83-2.02) mental health problems, lower cognitive performance, and adverse physical health.

**Conclusions:**

This study shows that an unbiased quantitative analysis of multidimensional SDOH can permit the determination of how SDOH patterns are associated with child developmental outcomes. Children exposed to socioeconomic deprivation showed the worst outcomes relative to other SDOH categories. These findings suggest the need to determine whether improvement in socioeconomic conditions can enhance child developmental outcomes.

## Key Points

**Question:**

What are the underlying patterns of the multidimensional social determinants of health (SDOH), and what are their associations with individual mental health, cognition, and physical health outcomes in children?

**Findings:**

In this cohort study of 10 504 children, significant disparities in child developmental health outcomes were observed across 4 patterns of SODH: (1) affluence, (2) high-stigma environment, (3) high socioeconomic deprivation, and (4) high crime and drug sale rates coupled with lower education and densely populated areas. The worst mental health, cognitive performance, and physical health outcomes were found in the high socioeconomic deprivation group (pattern 3).

**Meaning:**

The SDOH patterns analyzed in this study were able to capture and quantify the multidimensional nature of SDOH that children experience, and the finding that socioeconomic deprivation was associated with the worst outcomes should guide more targeted public health and social policies to address causes of child development disparities.

## Teaser

This cohort study identifies social determinants of health patterns and estimates their associations with US children’s mental health, cognition, and physical health.

## Introduction

Social determinants of health (SDOH), the “conditions in which people are born, grow, live, work, and age,”^1,2^ can affect health outcomes,^3,4,5^ including mental, cognitive, and physical well-being.^6,7^ Children are raised in neighborhoods with diverse SDOH profiles, including high poverty levels and unemployment; rural settings; limited access to quality health care, nutritious food, clean water, or educational opportunities; and increased exposure to crime and drug sales, and these SDOH are linked to an array of developmental problems, including mental health disorders,^7,8,9,10,11,12^ suicidal behaviors,^13,14,15^ cognitive performance,^6,7,16^ and physical health issues.^10,17^ Unraveling the complex relationships between SDOH and child development is crucial to understanding which SDOH combinations are associated with which developmental outcomes. Without such information, it is not possible to devise effective, targeted policies and interventions.

Current measurement tools are unable to capture the multidimensionality of SDOH. Most SDOH measures are composite indices, such as the Area Deprivation Index (ADI)^18^ and Social Vulnerability Index (SVI),^19^ that depend on an arbitrarily selected small set of SDOH variables and provide only generic and numeric vulnerability levels. These indices oversimplify the intricate clustering of SDOH and their potentially different effects on child health, forcing policymakers to attempt broad, and therefore more expensive, SDOH-focused interventions to mitigate health disparities. Moreover, previous research primarily correlates SDOH with broader county- and state-level outcomes^4,20,21^ instead of measuring individual-level child health directly,^13,14,15,22,23^ thereby increasing the risks of ecological fallacy.

The purpose of this study was to fill this critical knowledge gap by using innovative methodologies that capture and analyze the multidimensional nature of SDOH and uncover their associations with individual-level child mental health, cognition, and physical health outcomes. We hypothesized that distinct SDOH patterns exist, with different implications for child development and health outcomes.

## Methods

### Participants

This cohort study used data from the Adolescent Brain Cognitive Development (ABCD) Study, which recruited a diverse sample of children aged 9 to 10 years from 21 sites across the US, including urban, rural, and mountainous areas. The multistage probability sampling recruitment and oversampling for schools with more than 10% Black children by approximately 50% provided a close approximation to national sociodemographics based on age, sex, race and ethnicity, socioeconomic status, and urbanicity.^24^ Data were collected from September 1, 2016, to April 24, 2021.^25^ Institutional review boards at each study site approved the research protocol, with central institutional review board approval granted by the University of California, San Diego. All participants provided written informed consent.^26^ Children and parents completed surveys regarding child developmental outcomes and household socioeconomic characteristics. More details about recruitment and sampling strategies are described elsewhere.^25^ We followed the Strengthening the Reporting of Observational Studies in Epidemiology (STROBE) reporting guideline.

### Measures

#### SDOH Exposure

The ABCD Study linked population-level SDOH characteristics to children by using primary residential addresses.^27^ Therefore, the input measures are at the individual level. We systematically reviewed major SDOH conceptual frameworks from the World Health Organization,^1^ Healthy People 2030,^28^ the US Agency for Healthcare Research and Quality,^29^ and the US Centers for Disease Control and Prevention^2^ to select SDOH variables. We added 4 indicators regarding state-level implicit bias to account for structural stigma^27,30^ and variables used to construct common SDOH indices, including the Opportunity Atlas,^31^ Child Opportunity Index,^32^ ADI,^33,34,35^ and SVI,^19^ to be comparable with prior research.

**Figure 1.**
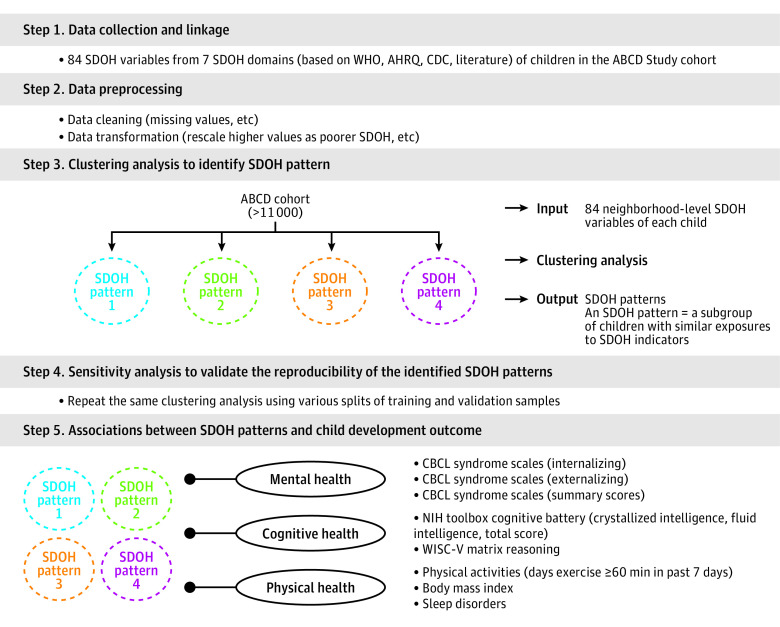
Study Pipeline ABCD, Adolescent Brain Cognitive Development; AHRQ, Agency for Healthcare Research and Quality; CBCL, Child Behavior Checklist; CDC, Centers for Disease Control and Prevention; NIH, National Institutes of Health; SDOH, social determinants of health; WHO, World Health Organization; WISC-V, Wechsler Intelligence Scale for Children.

We included 84 neighborhood-level SDOH variables across the following 7 SDOH domains (Figure 1; eMethods 1 and eTable 1 in Supplement 1): (1) bias (4 variables, eg, implicit attitudes toward race, explicit attitudes toward immigrant status), (2) education (11 variables, eg, availability of educational resources, number of early childhood centers), (3) physical and health infrastructure (16 variables, eg, percentage of mobile homes, crowded housing conditions), (4) natural environment (10 variables, eg, access to healthy food, walkability index), (5) socioeconomic status (27 variables, eg, poverty rate, residents without a high school diploma), (6) social context (8 variables, eg, proportion of people older than 65 years, people with disabilities), and (7) crime and drugs (11 variables, eg, county-level counts of arrests, state laws related to marijuana possession and use) (eMethods 1 in Supplement 1).

#### Child Mental Health, Cognitive Performance, and Physical Health 

All outcome measures are described in eMethods 2 in Supplement 1. We included measures of child psychopathology and suicidal ideation and behaviors assessed at baseline (wave 1) and 1-year (wave 2) and 2-year (wave 3) follow-up. Internalizing (eg, anxious, withdrawn, somatic complaints), externalizing (eg, rule breaking, aggressive behaviors), and social (eg, thoughts, social, attention) problems were assessed using the caregiver-reported Child Behavior Checklist (CBCL), which is an empirically validated and widely used assessment.^36,37,38,39^ We also included the CBCL *Diagnostic and Statistical Manual of Mental Disorders *(Fifth Edition) (*DSM-5*)–oriented symptom subscales for depression, anxiety, attention deficit hyperactivity disorder, somatic problems, opposition behaviors, and conduct problems. Raw scores on this measure were converted to t scores, with a t score >60 representing a clinically significant disorder.^36,37^ The computerized version of the Kiddie Schedule for Affective Disorders and Schizophrenia for *DSM-5* (Lifetime Version)^40^ was used to identify suicidal ideation and suicide attempts endorsed by either the child or caregiver.

The psychometrically sound National Institutes of Health Toolbox Cognition Battery^41^ was used to assess cognitive function in children,^42,43,44^ including 7 domain-specific cognitive tests from 2 categories (crystallized intelligence and fluid intelligence).^45,46,47,48,49^ We calculated the fully corrected crystallized cognition composite scores (derived from 2 subtests of the cognition battery: the picture vocabulary test and oral reading recognition test) and fluid cognition composite scores (derived from 5 subtests of the cognition battery: the card sort, flanker inhibitory control and attention test, picture sequence memory test, list-sorting working memory test, and pattern comparison processing speed test). We also used the Wechsler Intelligence Scale for Children (WISC-V),^50^ which measures nonverbal intelligence. We included 3 measures of physical health outcomes: body mass index (BMI), frequency of regular exercise for 60 minutes (days with ≥60-minute exercise in the past 7 days at survey), and sleep disorders (using the Sleep Disturbance Scale for Children,^51^ including subscores of 6 subdomains and composite score).

#### Covariates

We controlled for age, sex, and race and ethnicity reported in the baseline caregiver-completed ABCD Study Parent Demographics Survey. Race and ethnicity were based on baseline caregiver-reported categories from a list of provided terms and are included to account for the social construct that reflects youths’ experiences and exposure to social advantages and oppression.

### Statistical Analysis

#### Data Preprocessing and Missing Data

Among the 84 candidate SDOH variables for clustering analysis, 82 were continuous variables and 2 were categorical variables (converted to binary values). The percentage of missingness was small, ranging between 5% and 8% (eTable 1 in Supplement 1). Since data are not missing at random (eFigure 1 in Supplement 1), multiple imputations may not be reliable.^52^ Therefore, we only included children with complete SDOH data in the primary analysis to minimize bias. All continuous variables were *z* score scaled to eliminate artifactual effects caused by variables being measured on different scales. Finally, we obtained an SDOH variable vector for each child.

#### SDOH Pattern Identification

We applied the hierarchical agglomerative clustering algorithm to derive SDOH patterns using individual-level child SDOH vectors. The optimal cluster number was determined by considering cluster separation in the dendrogram, 14 cluster measurements using the NbClust package,^53^ and cluster separation in t-distributed stochastic neighbor embedding^54^ (eMethods 3 in Supplement 1). Each resultant cluster represents a specific SDOH pattern of individuals with similar SDOH profiles.

#### Sensitivity Analyses for SDOH Pattern Stability

We evaluated the stability of the identified SDOH patterns by conducting 3 sensitivity analyses (eMethods 4 in Supplement 1). First, we assessed whether excluding the 1306 children with any missing values from the analysis changed SDOH patterns. Second, we repeated the hierarchical agglomerative clustering and replicated the SDOH patterns using data from only the 9137 unique neighborhoods of residence for children in the sample, thereby avoiding any double counting of neighborhoods. Third, we conducted a robustness check of the study samples.

#### SDOH Pattern Interpretation

We visualized the resultant SDOH patterns using bar charts, depicting the proportion of disadvantaged children across different variables defined as exposure to more than 75% of the vulnerability level in the 82 continuous SDOH variables. For categorical variables, ie, marijuana law type and census tract urban classification, we used chord diagrams to illustrate their distribution across SDOH patterns.

We also compared baseline demographic, health data, and individual SDOH variables of children across the identified SDOH patterns, using analysis of variance tests (for continuous values) and χ^2^ tests (for categorical values). Multiple comparisons were adjusted using the Bonferroni correction. A 2-tailed *P* < .05 was considered the threshold for statistical significance.

#### Exploring Associations Between SDOH Patterns and Children’s Developmental Outcomes

We estimated the associations between the identified SDOH patterns and children’s health outcomes using mixed-effects linear regression models (for continuous outcomes) and mixed-effects logistic regression models (for binary outcomes). These models included study sites as a random effect to adjust the clustering design and age, sex, and race and ethnicity as covariates. We fitted models for mental health outcomes at baseline and 1-year and 2-year follow-up and for cognitive and physical outcomes at baseline due to data availability.

All statistical analyses were performed using Python, version 3.7 (Python Software Foundation) and R, version 4.1 (R Foundation for Statistical Computing) software. Clustering models were implemented based on Python packages scikit-learn, version 0.23.2 and scipy, version 1.5.3. The t-SNE algorithm was implemented based on the Python package scikit-learn, version 0.23.2. The R package NbClust was used to calculate measures of clusters to determine the optimal cluster number in hierarchical agglomerative clustering. Linear mixed-effects models and logistic mixed-effects models were fitted using lme4 in R. All other statistical tests were performed using R. Chord diagrams were created using the R package circlize. Other data visualization, including bar plots and forest plots, were generated using the Python package matplotlib, version 3.0.

## Results

### Descriptive Statistics

The ABCD Study cohort included 10 504 children (median [SD] age, 9.9 [0.6] years) with complete SDOH information. Of the studied participants, 5510 were boys (52.5%) and 4994 girls (47.5%), and 229 were Asian (2.2%), 1468 Black (14.0%), 2128 Hispanic (20.3%), 5565 White (53.0%), and 1108 multiracial (10.5%) (Table 1).

**Table 1.** Baseline Characteristics of the Study Cohort Across Social Determinants of Health (SDOH) Patterns

Characteristic^a^	All participants (N = 10 504)	SDOH pattern,^b^ No. (%)					
		1 (n = 4078)	2 (n = 2661)	3 (n = 2653)	4 (n = 1112)	*P* value^c^	Bonferroni-corrected *P* value
Age, mean (SD), y	9.9 (0.6)	9.9 (0.6)	9.9 (0.6)	9.9 (0.6)	9.9 (0.6)	<.001	.02
Sex							
Female	4994 (47.5)	1907 (46.8)	1250 (47.0)	1288 (48.5)	549 (49.4)	.29	>.99
Male	5510 (52.5)	2171 (53.2)	1411 (53.0)	1365 (51.5)	563 (50.6)		
Parents’ education (less than bachelor’s degree)	4176 (39.8)	766 (18.8)	981 (36.9)	1754 (66.1)	675 (60.7)	.01	.32
Race and ethnicity							
Asian	229 (2.2)	141 (3.5)	15 (0.6)	19 (0.7)	54 (4.9)	<.001	<.001
Black	1468 (14.0)	178 (4.4)	219 (8.2)	999 (37.7)	72 (6.5)		
Hispanic	2128 (20.3)	449 (11.0)	246 (9.2)	760 (28.6)	673 (60.5)		
White	5565 (53.0)	2890 (70.9)	1889 (71.0)	577 (21.7)	209 (18.8)		
Multiracial	1108 (10.5)	416 (10.2)	291 (10.9)	297 (11.2)	104 (9.4)		
Parents’ marital status							
Married	7177 (68.3)	3306 (81.1)	2035 (76.5)	1179 (44.4)	657 (59.1)	<.001	<.001
Single parent	2651 (25.2)	647 (15.9)	501 (18.8)	1181 (44.5)	322 (29.0)		
Living with partner	597 (5.7)	119 (2.9)	115 (4.3)	251 (9.5)	112 (10.1)		
Annual household income, $							
<50 000	2776 (26.4)	382 (9.4)	588 (22.1)	1356 (51.1)	450 (40.5)	<.001	<.001
50 000-100 000	2775 (26.4)	978 (24.0)	966 (36.3)	606 (22.8)	225 (20.2)		
>100 000	4082 (38.9)	2480 (60.8)	969 (36.4)	351 (13.2)	282 (25.4)		

^a^ Variables including participants’ demographic characteristics and family mental health history records were not used in clustering analysis for SDOH pattern identification.

^b^ Social determinants of health patterns: 1, affluent community; 2, high-stigma environment; 3, high socioeconomic deprivation; 4, high crime and drug sales, low education, high population density.

^c^ Comparisons across all 4 SDOH patterns were performed using analysis of variance for continuous variables and χ^2^ test for categorical variables. A 2-tailed *P* < .05 was considered as the threshold for statistical significance.

### Four Distinct SDOH Patterns

**Figure 2.**
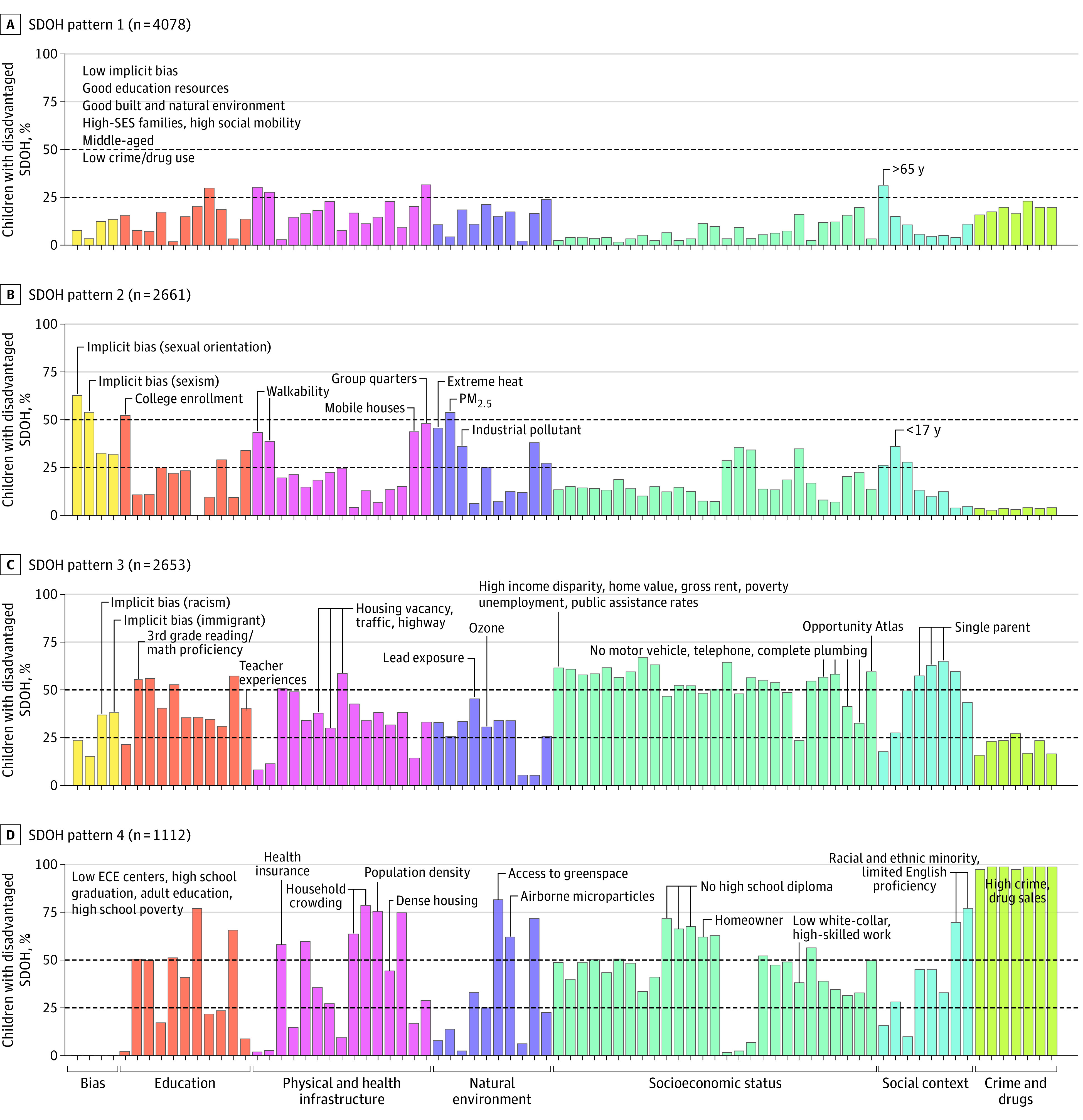
Characteristics of the Identified Social Determinants of Health (SDOH) Patterns Each bar shows the proportion of children with disadvantaged status in each (continuous) SDOH variable across SDOH patterns. Children with disadvantaged SDOH are those who have worse SDOH exposures than 75% of the population with regard to a specific SDOH variable. eFigure 4 in Supplement 1 provides more detailed information. ECE indicates early childhood education; SES, socioeconomic status.

Four child clusters were identified (eResults 1 and eFigures 2 and 3 in Supplement 1). Each cluster indicates a unique SDOH pattern (Figure 2; eFigures 4 and 5 and eTable 2 in Supplement 1).

#### Pattern 1

In SDOH pattern 1, 4078 children (38.8%) lived in affluent communities characterized by higher socioeconomic status, low structural stigma, moderate crime rates, and good academic performance (eg, highest third-grade reading and math proficiency). These children had easy access to healthy food, a healthier environment (eg, good air quality, low heat exposures), low social vulnerability (eg, lowest single-parent family rates, uncrowded housing, lowest minority populations), and moderate population density and walkability (Figure 2A).

#### Pattern 2

In SDOH pattern 2, 2661 children (25.3%) lived in a high-stigma environment that was marked by the highest implicit bias and discrimination toward women and sexual and gender minority groups and low college enrollment. These children lived in a poor environment for industrial pollutants, ozone, heat exposure, and particulate matter with a diameter less than 2.5 μm. The largest proportion of these children were aged 17 years or younger. This pattern included mobile homes and group quarters (eg, nursing homes, military barracks) and had the lowest number of white-collar workers, homeowners, and urbanicity characteristics (eg, lowest population density, lowest walkability, not close to highways) (Figure 2B).

#### Pattern 3

In SDOH pattern 3, 2653 children (25.3%) lived in a high socioeconomic deprivation environment that was characterized by the highest rates in most metrics of the ADI (eg, family income, income disparity, low home value, unemployment rates, high poverty rates, high single-family proportion, no telephone), SVI (eg, poverty rates, highest disabled population proportion), and Child Opportunity Index (eg, lowest health insurance, lowest reading and math proficiency, large public assistance–dependent family proportion) but the lowest Opportunity Atlas social mobility scores. Children in this pattern experienced the highest levels of racism and discrimination toward immigrants and the most severe lead exposure (Figure 2C).

#### Pattern 4

In SDOH pattern 4, 1112 children (10.6%) lived in environments characterized by high crime and drug sales, low education, and high population density. These children experienced the highest crime and drug sale rates, school poverty, air pollution (the highest amount of airborne microparticles), crowded housing, population density, and minority populations and the lowest levels of educational attainment and resources (eg, the lowest high school graduation rates and available early childhood education centers), access to a healthy environment (eg, lowest greenspace access), and homeownership rates (Figure 2D).

There are distinct racial and ethnic differences across SDOH patterns. Most White children were from affluent communities (pattern 1, 2890 [70.9%]) and high-stigma environments (pattern 2, 1889 [71.0]). Conversely, areas of high socioeconomic deprivation (pattern 3) revealed a much higher representation of Black (999 [37.7%]) and Hispanic (760 [28.6%]) children than their Asian counterparts (19 [0.7%]). Hispanic children were overrepresented in high-crime, low-education, and densely populated areas (pattern 4, 673 [60.5%]). Results of sensitivity analyses replicated the 4-cluster structure in the primary analyses and confirmed its robustness (eResults 2 and eFigures 6-8 in Supplement 1).

### Child Health Disparities Across SDOH Patterns

**Figure 3.**
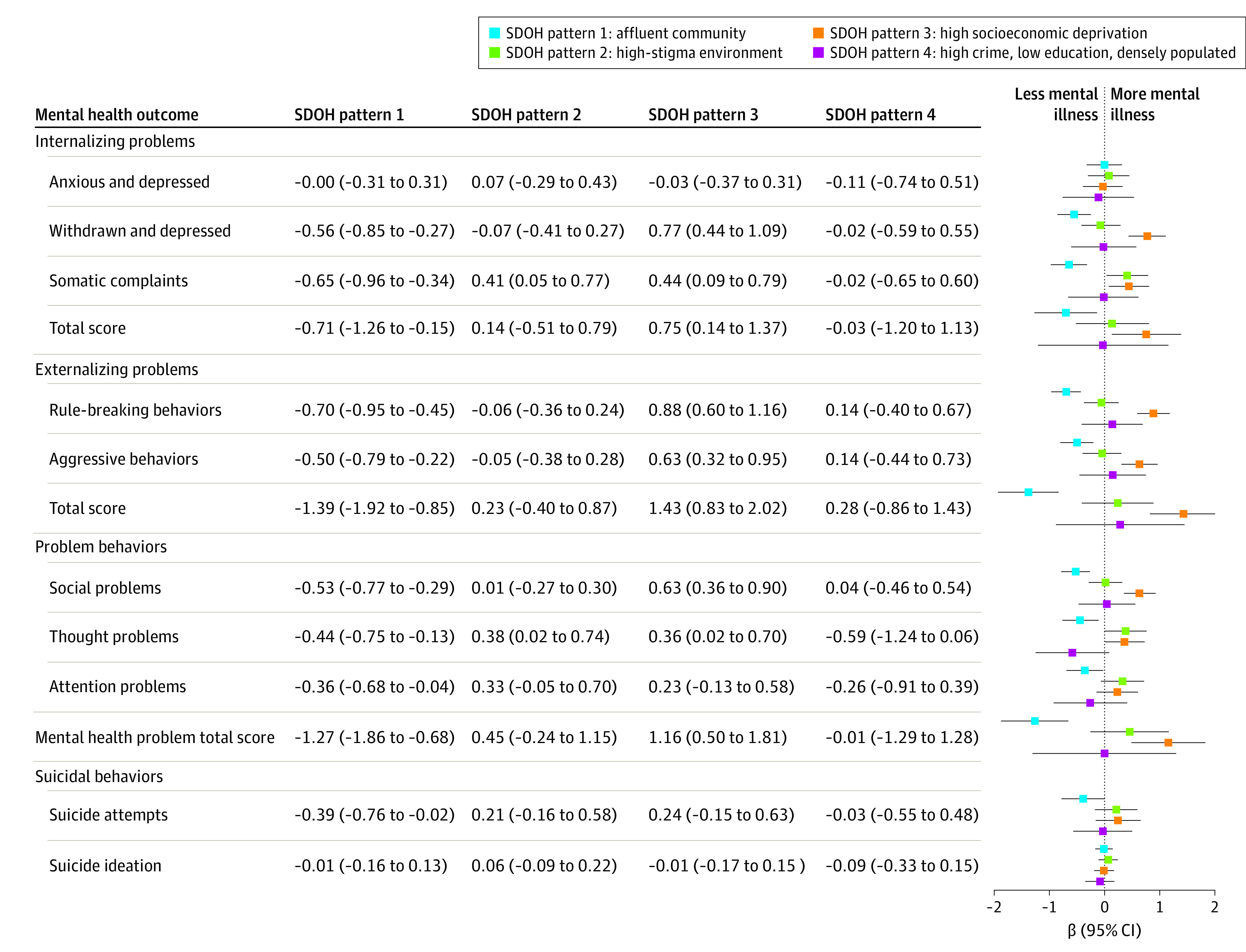
Associations Between the Identified Social Determinants of Health (SDOH) Patterns and Mental Health and Suicidal Behavior Outcomes of Children Child mental health outcomes were measured based on Child Behavior Checklist (CBCL) symptom scales (continuous)^36,37^ and self-reported suicidal behaviors using the self-administered computerized version of the Kiddie Schedule for Affective Disorders and Schizophrenia for *DSM-5* (Lifetime Version).^40^ A higher value on a CBCL symptom scale indicates worse mental illness of the child. For continuous outcomes (ie, CBCL symptom scales), β (95% CI) and *P* values were estimated based on linear mixed-effects regression analyses, adjusting for baseline age, sex, and race and ethnicity and including a random-effects term to account for within-site clustering. For binary outcomes (ie, suicidal behaviors), β (95% CI) and *P* values were estimated based on mixed-effects logistic regressions analyses, adjusting for baseline age, sex, and race and ethnicity and including a random-effects term to account for within-site clustering. *P* values can be found in eTable 3 in Supplement 1.

Significant differences in associated child developmental outcomes were found across the SDOH patterns (Table 2; Figure 3; eFigures 9-11 and eTable 3 in Supplement 1). Children living in SDOH pattern 3 (high socioeconomic deprivation) had the worst health outcomes relative to the other 3 SDOH patterns, including more mental health issues, suicidal behaviors, lower cognitive performance, and poor physical health (Table 2), even after adjusting for covariates (Figure 3; eFigure 11 and eTable 3 in Supplement 1).

**Table 2.** Baseline Mental Health, Cognitive, and Physical Health Outcomes Across Social Determinants of Health (SDOH) Patterns

Characteristic	All participants (N = 10 504)	SDOH pattern, mean (SD)^a^				*P* value	Bonferroni-corrected *P* value
		1 (n = 4078)	2 (n = 2661)	3 (n = 2653)	4 (n = 1112)		
**Mental health outcomes**							
CBCL internalizing problems							
Anxious and depressed	53.46 (5.91)	53.29 (5.69)	53.95 (6.56)	53.36 (5.72)	53.13 (5.40)	<.001	<.001
Withdrawn and depressed	53.47 (5.72)	53.06 (5.20)	53.62 (5.93)	54.01 (6.24)	53.30 (5.54)	<.001	<.001
Somatic complaints	54.89 (6.04)	54.42 (5.68)	55.44 (6.37)	55.18 (6.16)	54.61 (6.05)	<.001	<.001
Social problems	52.76 (4.68)	52.26 (4.20)	52.93 (4.91)	53.40 (5.15)	52.58 (4.37)	<.001	<.001
Total score	45.68 (10.27)	44.52 (9.77)	46.43 (10.21)	47.08 (10.96)	44.80 (9.91)	<.001	<.001
CBCL externalizing problems							
Rule-breaking behaviors	52.74 (4.83)	52.11 (4.13)	52.80 (4.77)	53.79 (5.80)	52.39 (4.38)	<.001	<.001
Aggressive behaviors	52.79 (5.46)	52.34 (4.87)	53.05 (5.70)	53.39 (6.16)	52.39 (4.94)	<.001	<.001
Total score	48.43 (10.59)	47.78 (10.24)	49.43 (10.78)	48.70 (10.83)	47.74 (10.58)	<.001	<.001
CBCL problem behaviors							
Thought problems	53.77 (5.86)	53.45 (5.48)	54.39 (6.28)	54.08 (6.27)	52.76 (4.86)	<.001	<.001
Attention problems	53.86 (6.11)	53.41 (5.69)	54.21 (6.43)	54.45 (6.56)	53.31 (5.51)	<.001	<.001
Total score	45.81 (11.26)	44.67 (10.75)	46.98 (11.10)	46.94 (11.95)	44.52 (11.20)	<.001	<.001
Suicidal behaviors, No (%)							
Suicide attempt	161 (1.5)	43 (1.1)	42 (1.6)	57 (2.1)	19 (1.7)	.004	.16
Suicide ideation	1308 (12.5)	515 (12.6)	348 (13.1)	318 (12.0)	127 (11.4)	.45	>.99
**Cognitive outcomes**							
Crystallized intelligence							
Picture vocabulary	52.34 (11.02)	53.60 (11.45)	51.62 (10.89)	50.91 (10.11)	52.89 (11.23)	<.001	<.001
Oral reading recognition	49.37 (11.58)	50.17 (11.74)	49.30 (11.61)	48.20 (11.05)	49.35 (11.94)	<.001	<.001
Total score	50.97 (11.28)	52.23 (11.59)	50.48 (11.18)	49.42 (10.58)	51.25 (11.48)	<.001	<.001
Fluid intelligence							
Flanker inhibitory control	46.06 (9.18)	46.37 (9.36)	45.38 (8.90)	45.99 (8.94)	46.78 (9.63)	<.001	<.001
List-sorting working memory	49.49 (9.95)	50.13 (9.73)	49.49 (9.49)	48.60 (10.36)	49.29 (10.68)	<.001	<.001
Dimensional change card sort	47.51 (9.69)	48.83 (10.44)	47.12 (9.23)	45.98 (8.72)	47.27 (9.44)	<.001	<.001
Pattern comparison processing speed	45.42 (14.27)	46.82 (14.28)	44.56 (13.69)	43.76 (14.58)	46.30 (14.37)	<.001	<.001
Picture sequence memory	49.56 (11.04)	49.84 (11.24)	49.64 (10.83)	48.93 (10.77)	49.87 (11.34)	.009	.10
Total score	45.87 (11.26)	47.13 (11.67)	45.16 (10.84)	44.42 (10.75)	46.46 (11.40)	<.001	<.001
Cognitive intelligence total score	47.81 (11.27)	49.40 (11.54)	47.10 (10.91)	45.90 (10.59)	48.30 (11.81)	<.001	<.001
WISC-V matrix reasoning total score	9.89 (2.97)	10.39 (2.80)	10.15 (2.87)	8.99 (3.03)	9.59 (3.16)	<.001	<.001
**Physical health outcomes**							
General physical health							
Days exercise ≥60 min in past 7 d	3.52 (2.31)	3.81 (2.22)	3.64 (2.37)	3.06 (2.33)	3.23 (2.26)	<.001	<.001
BMI	18.79 (4.23)	17.96 (3.47)	18.31 (3.91)	20.08 (4.94)	19.86 (4.70)	<.001	<.001
Sleep disorders scale							
Disorders of initiating and maintaining sleep	11.73 (3.72)	11.41 (3.50)	12.11 (4.03)	12.13 (3.83)	11.00 (3.22)	<.001	<.001
Sleep breathing disorders	3.76 (1.24)	3.61 (1.03)	3.73 (1.21)	4.00 (1.49)	3.78 (1.28)	<.001	<.001
Disorders of arousal	3.44 (0.92)	3.42 (0.80)	3.47 (0.96)	3.48 (1.09)	3.35 (0.75)	<.001	.002
Sleep-wake transition disorders	8.18 (2.61)	8.14 (2.49)	8.29 (2.71)	8.18 (2.73)	8.05 (2.53)	.04	.33
Disorders of excessive somnolence	6.94 (2.42)	6.75 (2.15)	7.05 (2.49)	7.23 (2.80)	6.65 (2.14)	<.001	<.001
Sleep hyperhidrosis	2.44 (1.19)	2.38 (1.03)	2.46 (1.25)	2.52 (1.32)	2.46 (1.25)	<.001	<.001
Total score	36.48 (8.19)	35.72 (7.14)	37.11 (8.71)	37.55 (9.25)	35.28 (7.32)	<.001	<.001

Abbreviations: BMI, body mass index (calculated as weight in kilograms divided by height in meters squared); CBCL, Child Behavior Checklist; WISC-V, Wechsler Intelligence Scale for Children.

^a^ Pattern 1, affluent community; pattern 2, high-stigma environment; pattern 3, high socioeconomic deprivation; pattern 4, high crime and drug sales, low education, high population density.

**Figure 4.**
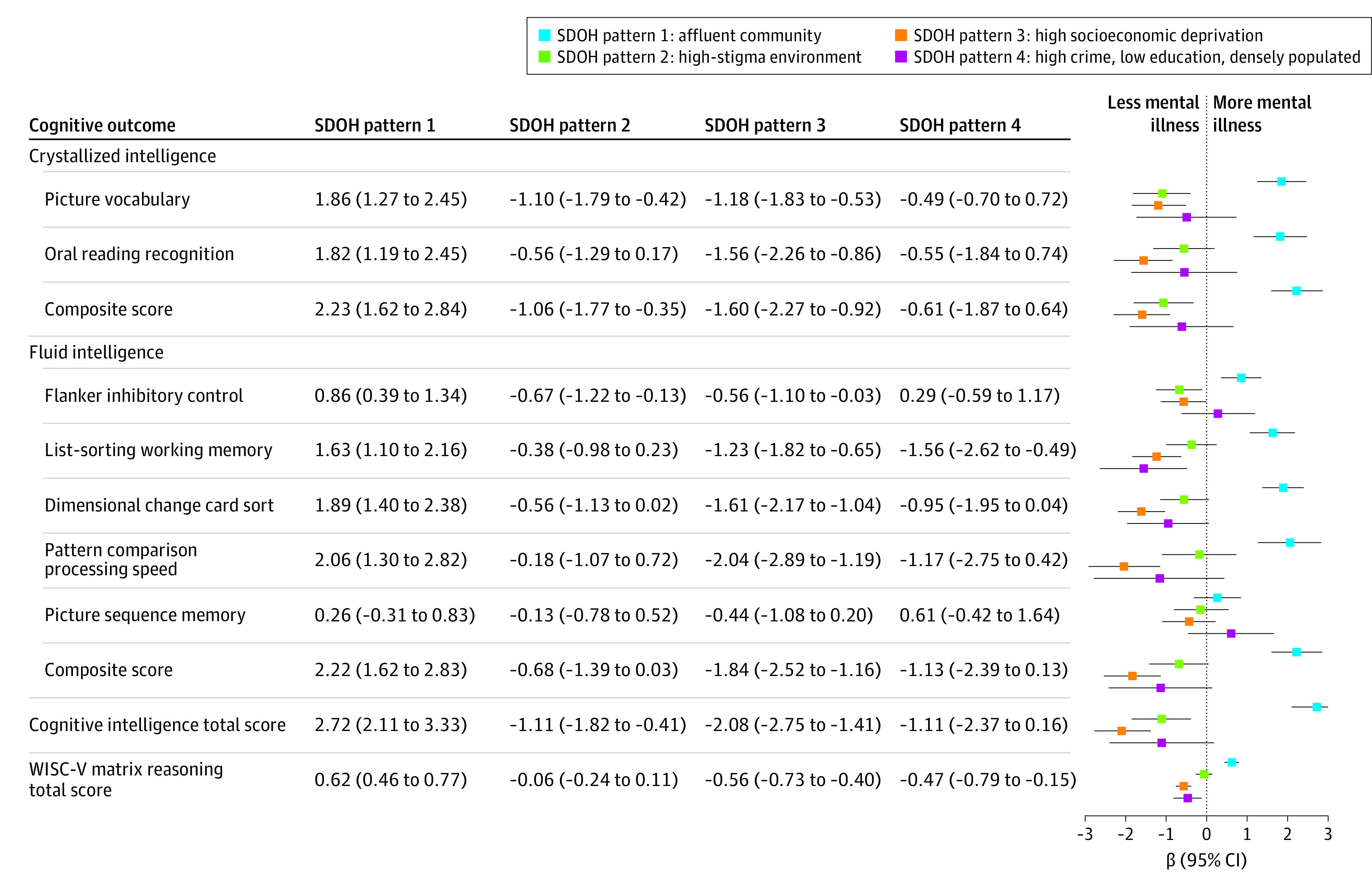
Associations Between the Identified Social Determinants of Health (SDOH) Patterns and Cognitive Outcomes of Children Cognitive intelligence total score was calculated as the summation of crystallized intelligence and fluid intelligence scores for children. For each score, a higher value indicates better cognitive health of the child. For continuous outcomes (ie, CBCL symptom scales), β (95% CI) and *P* values were estimated based on linear mixed-effects regression analyses, adjusting for baseline age, sex, and race and ethnicity and including a random-effects term to account for within-site clustering. For binary outcomes (ie, suicidal behaviors), β (95% CI) and *P* values were estimated based on mixed-effects logistic regressions analyses, adjusting for baseline age, sex, and race and ethnicity and including a random-effects term to account for within-site clustering. *P* values can be found in eTable 3 in Supplement 1.

Specifically, children living in SDOH pattern 3 had the most severe internalizing problems (β = 0.75; 95% CI, 0.14-1.37; *P* = .02), externalizing problems (β = 1.43; 95% CI, 0.83-2.02; *P* < .001), social problems (β = 0.63; 95% CI, 0.36-0.90; *P* < .001), and mental health problems (ie, CBCL total score: β = 1.16; 95% CI, 0.50-1.81; *P* < .001) relative to the other 3 SDOH patterns (Figure 3; eTable 3 in Supplement 1). In addition, SDOH pattern 3 was associated with children’s poorer cognitive performance (Figure 4; eTable 3 in Supplement 1), including lower crystallized intelligence (β = −1.60; 95% CI, −2.27 to −0.92; *P* < .001), fluid intelligence (β = −1.84, 95% CI, −2.52 to −1.16; *P* < .001), cognitive intelligence total score (β = −2.08; 95% CI, −2.75 to −1.41; *P* < .001), and WISC-V reasoning score (β = −0.56; 95% CI, −0.73 to −0.40; *P* < .001). Furthermore, these children were the least active, as evidenced by a lower probability of exercising 60 minutes in the past 7 days (β = −0.33; 95% CI, −0.46 to −0.20; *P* < .001), a higher BMI (β = 1.01; 95% CI, 0.77-1.25; *P* < .001), and overall sleep disorder problems (β = 0.77; 95% CI, 0.29-1.25; *P* = .002) (eFigure 11 and eTable 3 in Supplement 1).

Conversely, children residing in SDOH pattern 1 (affluent communities) had better developmental outcomes, including fewer mental health issues, fewer suicide attempts, higher cognitive scores, and better physical health (Figure 3, Figure 4; eFigure 11 and eTable 3 in Supplement 1). After accounting for covariates, SDOH pattern 1 was associated with fewer internalizing problems (β = −0.71; 95% CI, −1.26 to −0.15; *P* = .01), externalizing problems (β = −1.39; 95% CI, −1.92 to −0.85; *P* < .001), social problems (β = −0.53; 95% CI, −0.77 to −0.29; *P* < .001), thought problems (β = −0.44; 95% CI, −0.75 to −0.13; *P* = .005), attention problems (β = −0.36; 95% CI, −0.68 to −0.04; *P* = .03), and overall mental health problems (β = −1.27; 95% CI, −1.86 to −0.68; *P* < .001) and a lower likelihood of suicide attempts (β = −0.39; 95% CI, −0.76 to −0.02; *P* = .04) (Figure 3; eTable 3 in Supplement 1). Cognitively, children in SDOH pattern 1 showed the best cognitive performances (Figure 4), including crystallized intelligence (β = 2.23; 95% CI, 1.62-2.84; *P* < .001), fluid intelligence (β = 2.22; 95% CI, 1.62-2.83; *P* < .001), cognitive intelligence total score (β = 2.72; 95% CI, 2.11-3.33; *P* < .001), and WISC-V reasoning score (β = 0.62; 95% CI, 0.46-0.77; *P* < .001). Physically, children in SDOH pattern 1 engaged in more 60-minute exercise (β = 0.31; 95% CI, 0.19-0.42; *P* < .001), had lower BMIs (β = −0.97; 95% CI, −1.19 to −0.76; *P* < .001), and had a lower sleep disorders total score (β = −0.71; 95% CI, −1.14 to −0.28; *P* < .001) (eFigure 11 and eTable 3 in Supplement 1).

Children living in a pattern 2 (high stigma) or pattern 3 (high crime and drug sales, low education, and high population density) environment exhibited lower cognitive abilities (SDOH pattern 2, cognitive intelligence total score: β = −1.11 [95% CI, −1.82 to −0.41; *P* = .002]; SDOH pattern 4, WISC-V score: β = −0.47 [95% CI, −0.79 to −0.15; *P* = .005]) relative to the other patterns (Figure 4), with children in SDOH pattern 4 also showing lower levels of physical activity (β = −0.22; 95% CI, −0.45 to 0.01; *P* = .06), high BMI (β = 1.01; 95% CI, 0.54-1.47; *P* < .001), and worse sleep quality (β = −0.93; 95% CI, −1.83 to −0.02; *P* = .046) than the other 3 SDOH patterns (eFigure 11 and eTable 3 in Supplement 1).

## Discussion

Our cohort study used machine learning techniques to identify multifaceted SDOH patterns from a wide spectrum of 84 population-level SDOH indicators and estimated their associations with child development. Children in areas of high socioeconomic deprivation experienced the worst developmental outcomes, while those living in affluent areas reported better mental health, cognitive, and physical health outcomes. Additionally, children residing in high-stigma environments and areas characterized by higher crime rates and drug sales, lower education attainment, and higher population density had lower cognitive performance (cognitive intelligence, reasoning scores), higher BMI, and more sleep disorders, underscoring the association of heterogeneous outcomes of SDOH with child development.

Our approach transcends the traditional scales or single-index methods that predominate in the literature but have been incapable of capturing the interconnected and multidimensional SDOH and associating them with specific health and developmental outcomes. We estimated the collective influence of co-occurring SDOH patterns on child development, setting a foundation for more precise, targeted health care policy planning and implementation.^55^

Our SDOH findings reflect distinct racial and ethnic differences. White children were overrepresented in affluent and high-stigma environments, whereas Black and Hispanic children were overrepresented in socioeconomically deprived areas, derived from discriminatory housing policies and resource accessibility^56^ and reflecting systemic racism and socioeconomic marginalization that restrict Black and Hispanic children’s access to opportunities.^57^ The higher rates of crime, low education, and dense populations in areas populated by Hispanic and Black residents may influence the mental, cognitive, and physical health of these children, as suggested by prior studies.^58,59,60^ These findings suggest, but are not proof, that socioeconomic deprivation may have a detrimental association with many child developmental outcomes.^61^ Factors such as restricted access to educational resources and cognitive stimulation, poor nutrition, limited opportunities for physical activity, and exposure to environmental stressors, may potentially disrupt sleep patterns,^62,63,64,65^ worsen mental health, and increase the risk of suicide attempts.^66^ Compared with other SDOH patterns, children living in highly stigmatizing environments exhibited lower cognitive abilities consistent with the possibility that chronic stress^67^ (which alters hypothalamic-pituitary-adrenal axis function and increases cortisol levels^68^), limited access to quality education,^69^ and inadequate health care and nutrition can negatively affect brain development and cognitive function.^70^

Children living in areas characterized by high crime and drug sales, low education, and high population density were observed to exhibit both lower cognitive function and insufficient physical activity compared with the other SDOH patterns. The chronic stress in these communities,^71,72^ coupled with limited access to basic resources,^73^ can hinder cognitive development and discourage physical activity, deteriorating overall cognition and activity levels.

### Strengths and Limitations

The strengths of this study lie in the use of a data-driven approach, facilitating the identification of nuanced high-dimensional patterns of SDOH and their varied associations with child development. Our measures of child developmental outcomes are comprehensive and consistent with the gold standards of child mental, cognitive, and physical health assessments. We focused on linking area-based SDOH to individual-level outcomes, thereby minimizing the likelihood of ecological fallacy.^74,75,76,77^

However, the study also has limitations. First, outcome measures were self-reported and may be biased by underreporting or discrepancies in reports by the child or parents.^78^ Second, the ABCD Study sample is not nationally representative, covering 17 states across 21 study sites. Consequently, our results may not apply to all US children. Third, our SDOH data, constrained by the available ABCD-linked external data, may not cover all potential SDOH variables. However, the selection of the 84 SDOH variables is theoretically^79,80,81,82^ and empirically^80^ sound. Additionally, we focused on bias and stigma calculated using objective state-level indicators of social policies and prejudicial attitudes, with the goal of capturing broad societal and institutional biases. Such measures were not intended to consider individual-level bias derived from self-reported discrimination. Consequently, we may have overlooked influences arising from personal bias experiences and their interaction with structural factors. Fourth, there were missing data for some variables in the clustering models. Despite these limitations, our study contributes valuable insights and prompts further investigation into the role of SDOH in children’s health outcomes.

## Conclusions

 In this cohort study, we derived 4 SDOH patterns that quantify and express multidimensional SDOH and then linked these patterns to differences in child development and health outcomes. By linking specific outcomes to specific SDOH patterns, our findings emphasize the need for targeted interventions to tackle potential causes of adverse health and developmental outcomes, especially in children with greater socioeconomic deprivation. A holistic approach involving efforts of policymakers and health care practitioners is crucial to nurturing child resilience and promoting health equity.

## Notes

Supplement 1. **eMethods 1.** Social Determinants of Health Variables **eMethods 2.** Detailed Measures of Study Outcomes **eMethods 3.** Determination of the Optimal Cluster Number **eMethods 4.** Sensitivity Analyses **eResults 1.** Determination of the Optimal Number of Clusters (ie, SDOH Patterns) in the Primary Analysis **eResults 2.** Sensitivity Analysis to Validate the Stability and Reproducibility of SDOH Patterns
eReferences
**eFigure 1.** An Illustration of Missing Values in the Child × SDOH Variable Matrix **eFigure 2.** Dendrogram for SDOH Pattern Identification in the Primary Analysis **eFigure 3.** Visualization of SDOH Patterns in 2D t-SNE Space **eFigure 4.** Characteristics of the Identified Social Determinants of Health (SDOH) Patterns (With Detailed Information) **eFigure 5.** Association Between SDOH Patterns With Marijuana State Law Types and Census Tract Urban Classification **eFigure 6.** Results of Sensitivity Analysis to Dropped Samples Due to Missing Values **eFigure 7.** Results of Sensitivity Analysis at the Neighborhood Level **eFigure 8.** Results of Sensitivity Analysis to Include Data Samples **eFigure 9.** Forest Plot Showing Associations Between the Identified Social Determinants of Health (SDOH) Patterns and Mental Health and Suicidal Behavior Outcomes of Children at 1-Year Follow-Up **eFigure 10.** Forest Plot Showing Associations Between the Identified Social Determinants of Health (SDOH) Patterns and Mental Health and Suicidal Behavior Outcomes of children at 2-Year Follow-Up **eFigure 11.** Forest Plot Showing Associations Between the Identified Social Determinants of Health (SDOH) Patterns and Physical Health Outcomes of Children **eTable 1.** The SDOH Variables Used for SDoH Pattern Identification **eTable 2.** Characteristics in SDOH Variables by the Identified SDOH Patterns **eTable 3.** *P* Values of Associations Between the Identified SDOH Patterns and Mental Health, Cognition, and Physical Health Outcomes

Supplement 2.
Data Sharing Statement
