# Supplementary material for: Patterns of Social Determinants of Health and Child Mental Health, Cognition, and Physical Health
Source: JAMA Pediatr. 2023 Oct 16;177(12):1294–305. doi: 10.1001/jamapediatrics.2023.4218 (PMC10580157; doi:10.1001/jamapediatrics.2023.4218)
Supplement: Supplement 1. — eMethods 1. Social Determinants of Health Variables eMethods 2. Detailed Measures of Study Outcomes eMethods 3. Determination of the Optimal Cluster Number eMethods 4. Sensitivity Analyses eResults 1. Determination of the Optimal Number of Clusters (ie, SDOH Patterns) in the Primary Analysis eResults 2. Sensitivity Analysis to Validate the Stability and Reproducibility of SDOH Patterns eReferences eFigure 1. An Illustration of Missing Values in the Child × SDOH Variable Matrix eFigure 2. Dendrogram for SDOH Pattern Identification in the Primary Analysis eFigure 3. Visualization of SDOH Patterns in 2D t-SNE Space eFigure 4. Characteristics of the Identified Social Determinants of Health (SDOH) Patterns (With Detailed Information) eFigure 5. Association Between SDOH Patterns With Marijuana State Law Types and Census Tract Urban Classification eFigure 6. Results of Sensitivity Analysis to Dropped Samples Due to Missing Values eFigure 7. Results of Sensitivity Analysis at the Neighborhood Level eFigure 8. Results of Sensitivity Analysis to Include Data Samples eFigure 9. Forest Plot Showing Associations Between the Identified Social Determinants of Health (SDOH) Patterns and Mental Health and Suicidal Behavior Outcomes of Children at 1-Year Follow-Up eFigure 10. Forest Plot Showing Associations Between the Identified Social Determinants of Health (SDOH) Patterns and Mental Health and Suicidal Behavior Outcomes of children at 2-Year Follow-Up eFigure 11. Forest Plot Showing Associations Between the Identified Social Determinants of Health (SDOH) Patterns and Physical Health Outcomes of Children eTable 1. The SDOH Variables Used for SDoH Pattern Identification eTable 2. Characteristics in SDOH Variables by the Identified SDOH Patterns eTable 3. P Values of Associations Between the Identified SDOH Patterns and Mental Health, Cognition, and Physical Health Outcomes [file jamapediatr-e234218-s001.pdf]

Supplemental Online Content

Xiao Y, Mann JJ, Chow JC-C, et al. Patterns of social determinants of health and child mental health, cognition, and physical health. *JAMA Pediatr*. Published online October 16, 2023. doi:10.1001/jamapediatrics.2023.4218

eMethods 1. Social Determinants of Health Variables

eMethods 2. Detailed Measures of Study Outcomes

eMethods 3. Determination of the Optimal Cluster Number

eMethods 4. Sensitivity Analyses

eResults 1. Determination of the Optimal Number of Clusters (ie, SDOH Patterns) in the Primary Analysis

eResults 2. Sensitivity Analysis to Validate the Stability and Reproducibility of SDOH Patterns

eReferences

eFigure 1. An Illustration of Missing Values in the Child × SDOH Variable Matrix

eFigure 2. Dendrogram for SDOH Pattern Identification in the Primary Analysis

eFigure 3. Visualization of SDOH Patterns in 2D t-SNE Space

eFigure 4. Characteristics of the Identified Social Determinants of Health (SDOH) Patterns (With Detailed Information)

eFigure 5. Association Between SDOH Patterns With Marijuana State Law Types and Census Tract Urban Classification

eFigure 6. Results of Sensitivity Analysis to Dropped Samples Due to Missing Values

eFigure 7. Results of Sensitivity Analysis at the Neighborhood Level

eFigure 8. Results of Sensitivity Analysis to Include Data Samples

eFigure 9. Forest Plot Showing Associations Between the Identified Social Determinants of Health (SDOH) Patterns and Mental Health and Suicidal Behavior Outcomes of Children at 1-Year Follow-Up

eFigure 10. Forest Plot Showing Associations Between the Identified Social Determinants of Health (SDOH) Patterns and Mental Health and Suicidal Behavior Outcomes of children at 2-Year Follow-Up

eFigure 11. Forest Plot Showing Associations Between the Identified Social Determinants of Health (SDOH) Patterns and Physical Health Outcomes of Children

eTable 1. The SDOH Variables Used for SDOH Pattern Identification

eTable 2. Characteristics in SDOH Variables by the Identified SDOH Patterns

eTable 3. *P* values of associations between the identified SDOH Patterns and mental health, cognition, and physical health outcomes.

This supplemental material has been provided by the authors to give readers additional information about their work.

© 2023 American Medical Association. All rights reserved.

eMethods 1. Social Determinants of Health Variables

We included 84 neighborhood-level SDOH variables across seven SDOH domains as below.

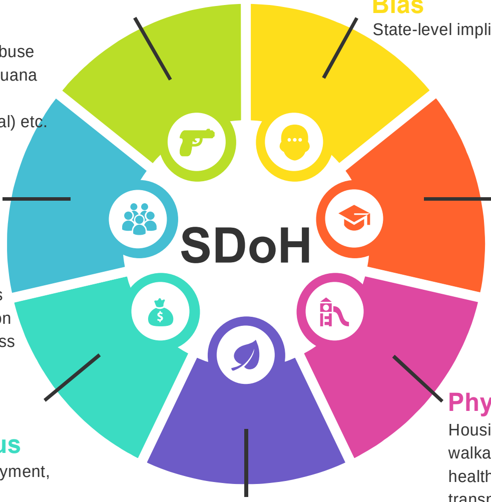

Crime & Drugs
Uniform Crime Reports: Total adult offense, adult violent crimes, drug abuse violations total, drug sale total, Marijuana sale, drug possession total, DUI, Marijuana laws (recreational, medical) etc.

Bias
State-level implicit bias for sexism, racism, sexual orientation, immigrants

Social Context
% of persons at least 65 years old, <17 years, civilian non-institutionalized population, single-parent households with children less than 18 years old, minority population (i.e., all but white, non-Hispanic), less English proficiency, etc.

Education
ECE Centers, High-quality ECE Centers, ECE enrollment, 3rd grade reading/math proficiency, High school graduation rate, Advanced Placement (AP) enrollment, Nearby college enrollment, School poverty, Teacher experience, Adult educational attainment, etc.

Physical & Health Infrastructure
Housing density, crowding, traffic, housing vacancy rate, walkability, access to green space, access to highway, access to healthy food, available social services, access to public transportation, etc.

Socioeconomic Status
% of income, education, unemployment, home values, mortgage etc.

Natural Environment
Hazardous waste dump sites, Industrial pollutants in air, water or soil, Airborne microparticles, Ozone concentration, Extreme heat exposure, etc.

Construction of the Structural Bias/Stigma Variable

The bias variables are previously validated and modeled as indicators in factor analysis.[1] The end product was a factor score, which determined the structural stigma score for each state across various stigma domains. Except for anti-immigrant sentiment, where available indicators were limited, all indicators used had measurements for every state, including Washington, D.C (as opposed to limiting to the 17 states where the ABCD sample were recruited). The inclusion of all 50 states was crucial to situate the factor scores within the nation's overall distribution. Model-based factor scores are centered around a mean of 0 in a normal distribution.

Every measure was coded to indicate that higher levels represented greater structural stigma. These measures were standardized to the average response value for all respondents, irrespective of their state of residence, and were then aggregated at the state level. As a result, each state's measure was the mean of the standardized individual responses for respondents living in that state. This rule did not apply to state-level variables, which were already at the state level and therefore required no further aggregation or standardization. All measures were included in the model selection if they represented residents from every state, including Washington, D.C. However, anti-immigrant sentiment, which depended heavily on state policy measures, did not include data from Washington, D.C., and was hence excluded from the models.

All responses at the state level were aggregated, regardless of the year of the survey. This process of averaging the responses enabled all states to have a substantial number of respondents, despite year-to-year sampling variation, thereby minimizing measurement error. Moreover, previous studies have indicated that while structural sexism and racism have seen a national decrease over time, the relative stigma levels of individual states (that is, rankings in relation to other states) have remained consistent. This suggests that a time-invariant measure is a valid method of operationalizing this construct.

For more information, readers can refer to the original article describing the process[1].

© 2023 American Medical Association. All rights reserved.

eMethods 2. Detailed Measures of Study Outcomes

Child Mental Health Outcomes

We used the CBCL-generated summary scores for three sub-categories—internalizing, externalizing, and total problems—and scores for eight individual dimensions within these sub-categories. Raw scores of these scales were converted to t-scores using sex- and age-based norms from population-based studies. Higher scores indicated a greater prevalence of problems., with a t-score greater than 60 representing probable disorder.
The analyzed CBCL scores include:

- (Internalizing problems)
  - Anxious/Depressed scale
  - Withdrawn/Depressed scale
  - Somatic Complaints scale
  - Internalizing Problems Total Score
- (Externalizing problems)
  - Rule-Breaking Behavior scale
  - Aggressive Behavior scale
  - Externalizing Problems Total Score
- (Problem behaviors)
  - Social Problems scale
  - Thought Problems scale
  - Attention Problems scale
- Mental Health Problem Total scale

We used self-report suicidal behaviors, including

- Suicidal ideation
- Suicidal attempts

Child Cognitive Health Outcomes

We used NIH Toolbox Cognition Battery (CB) individual items and composite scores, including

- (Crystallized intelligence)
  - Picture Vocabulary Test (asks participants to pair pictures with audible descriptions)
  - Oral Reading Recognition Test (pronunciation of single words)
  - Crystallized Intelligence Composite score
- (Fluid intelligence)
  - Flanker Inhibitory Control and Attention Test (inhibition of attention to irrelevant aspects of a task by presenting a central target with flanking stimuli to the left and right)
  - List-Sorting Working Memory Test (assess the reproduction of visually and orally presented test items in order of size)
  - Dimensional Change Card Sort Test (a measure of cognitive flexibility that involves matching a target stimulus to 1 dimension, then switching to match the target stimulus to another dimension)
  - Pattern Comparison Processing Speed Test (measures the speed of processing by asking participants to discern whether 2 side-by-side pictures are the same or different)
  - Picture Sequence Memory Test (presentation of a set of pictures in a fixed order accompanied by a verbal description, which the participant must then remember and reproduce)
  - Fluid Intelligence Composite score
- Cognitive Intelligence Composite score

The battery is psychometrically sound, incorporating item response theory and computerized adaptive testing, and is based on normative data from 5,000 participants as part of the NIH Blueprint for Neuroscience Research. The battery is psychometrically sound, incorporating item response theory and computerized adaptive testing, and is based on normative data from 5,000 participants as part of the NIH Blueprint for Neuroscience Research.[2] The NIH Toolbox CB includes seven domain-specific cognitive tests from two categories (crystallized intelligence and fluid intelligence) with good convergent validity compared with existing gold standards of cognitive testing. [3-6] We used the seven NIH Toolbox CB individual items, crystallized intelligence and fluid intelligence composite scores, cognitive function total score (derived using scores from all seven test domains).

© 2023 American Medical Association. All rights reserved.

We also used the Wechsler Intelligence Scale for Children-Fifth Edition (WISC-V).[7]

Child Physical Health Outcomes

We used children's general physical health outcomes and sleep disorder outcomes measured by the Sleep Disturbance Scale for Children [SDSC][8] sub-items and composite score, including:

- (General physical health)
  - Body mass index (BMI)
  - Days with ≥60-minute exercise in the past 7 days at survey (frequency of regular exercise for 60 minutes)
- (Sleep disorders)
  - Disorders of Initiating and Maintaining Sleep
  - Sleep Breathing Disorders
  - Disorders of Arousal
  - Sleep-Wake Transition Disorders
  - Disorders of Excessive Somnolence
  - Sleep Hyperhidrosis
  - Sleep Disorder Composite scale

© 2023 American Medical Association. All rights reserved.

eMethods 3. Determination of the Optimal Cluster Number

We used the hierarchical agglomerative clustering (HAC) algorithm on children's SDOH variable vectors to derive SDOH patterns. We used the HAC algorithm because it is robust to different types of data distributions and typically produces a tree-like diagram known as a dendrogram, which visualizes how the data points are agglomerated together in a hierarchical manner, helping determine optimal cluster number. We used a HAC algorithm with a Euclidean distance calculation based on children's SDOH variable vectors. The Ward linkage criterion[9] was used to construct the hierarchy for identifying the cluster structure.

To determine the optimal number of clusters, we considered three criteria as follows.

1. The hierarchical agglomerative clustering (HAC) algorithm typically generates a dendrogram, a tree-like diagram that shows how clusters are merged step-by-step. The clusters should be clearly separated in the dendrogram produced by the HAC. One straightforward way to determine clusters is to visually inspect the dendrogram. The clusters correspond to the vertical lines on the dendrogram, and the length of the lines represents the distance (dissimilarity) between clusters. The longer the line (i.e., the greater the distance), the less similar the merged clusters are.

2. We measure clustering performance using 14 indices in 'NbClust', including Krzanowski-Lai index, Calinski-Harabasz index, Hartigan index, C-index, Davies-Bouldin index, Ratkowsky index, Ball index, Ptbiserial index, Gap index, Frey index, McClain index, Dunn index, SD index, and SDbw index.

3. We calculate the 2-dimensional (2D) representation for each child based on the 84 SDOH variables for clustering using t-distributed stochastic neighbor embedding (t-SNE). We then visualize children's SDOH pattern memberships in the 2D space. We expect that the clusters could be clearly separated in the 2D t-SNE space.

© 2023 American Medical Association. All rights reserved.

eMethods 4. Sensitivity Analyses

We conducted three sensitivity analyses to assess the stability of our identified clusters (i.e., SDOH patterns).

1. **Sensitivity to dropping samples due to missing values.** We re-analyzed the data by including children whose SDOH variables are with a missing rate of < 70%. Like the primary analysis, the SDOH variables were scaled based on z-score. K-nearest neighbors (KNN) imputation[10] was used to address missing values. After that, cluster analysis was conducted to re-identify SDOH patterns.

2. **Sensitivity analysis at the neighborhood level.** Since we don't have geographical information of the participants, we considered that children with the same SDOH profiles are from the same neighborhood. The 10,504 children included in the primary analysis are residing in 9,137 different neighborhoods. We remove duplicated samples within each neighborhood and re-conducted cluster analysis.

3. **Sensitivity to the study sample**. Specifically, following our previous study,[11] we randomly split the entire cohort into 5 folds. Then each time, we successively dropped 1 fold (20% of the sample) and used the remaining 4 folds (80% of the sample) to construct a subset. We then re-identified SDOH patterns in each subset.

Of note, in these sensitivity analyses, SDOH patterns were re-identified using the HAC algorithm, following the same criteria as in the primary analysis for determining the optimal number of clusters. We compared the re-identified SDOH patterns with those identified in the primary analysis presented in the main text. Cluster labels from the primary analysis and the sensitivity analyses were aligned based on a manual review of the SDOH profiles of the clusters. We then assessed for overlap between the SDOH patterns derived in the primary analysis with those derived in the sensitivity analyses.

© 2023 American Medical Association. All rights reserved.

eResults 1. Determination of the Optimal Number of Clusters (ie, SDOH Patterns) in the Primary Analysis

In the primary analysis, we took several steps to determine the optimal number of clusters, (i.e., SDOH patterns) based on the multidimensional, heterogenous SDOH data. Each SDOH pattern represents a cluster of children exposed to similar patterns of 84 SDOH indicators across the 7 domains.

Initially, the 'NbClust' algorithm suggested the presence of three potential clusters, as deduced by majority voting across 14 cluster measurements. However, to prevent a single SDOH pattern from dominating the study population, we took the largest cluster (which comprised over 64% of the children in our cohort) and split it into two separate clusters. This process led us to identify 4 distinct SDOH patterns or clusters.

The 4-cluster structure was further corroborated by a dendrogram (eFigure 2), generated using a hierarchical clustering algorithm, and a 2D t-SNE plot (eFigure 3).

Hence, the optimal cluster (SDOH pattern) number was 4 in the primary analysis.

© 2023 American Medical Association. All rights reserved.

eResults 2. Sensitivity Analysis to Validate the Stability and Reproducibility of SDOH Patterns

In all sensitivity analyses, the HAC algorithm still detected the 4-cluster structure (i.e., 4 SDOH patterns), and the same as what were identified in the primary analysis. The re-identified clusters (i.e., SDOH patterns) were highly overlapped with that identified in the primary analysis. (See eFigures 6-8.)

© 2023 American Medical Association. All rights reserved.

eReferences

1. Hatzenbuehler ML, Weissman DG, McKetta S, et al. Smaller Hippocampal Volume Among Black and Latinx Youth Living in High-Stigma Contexts. *Journal of the American Academy of Child & Adolescent Psychiatry.* 2022;61(6):809-819.
2. Mott MC, Austin CP, Bianchi DW, et al. The NIH Blueprint for neuroscience research seeks community input on future neuroscience investments. *The Journal of Neuroscience.* 2019;39(5):774.
3. Luciana M, Bjork JM, Nagel BJ, et al. Adolescent neurocognitive development and impacts of substance use: Overview of the adolescent brain cognitive development (ABCD) baseline neurocognition battery. *Developmental Cognitive Neuroscience.* 2018;32:67-79.
4. Zelazo PD, Anderson JE, Richler J, Wallner-Allen K, Beaumont JL, Weintraub S. II. NIH TOOLBOX COGNITION BATTERY (CB): MEASURING EXECUTIVE FUNCTION AND ATTENTION. *Monographs of the Society for Research in Child Development.* 2013;78(4):16-33.
5. Gershon RC, Slotkin J, Manly JJ, et al. IV. NIH TOOLBOX COGNITION BATTERY (CB): MEASURING LANGUAGE (VOCABULARY COMPREHENSION AND READING DECODING). *Monographs of the Society for Research in Child Development.* 2013;78(4):49-69.
6. Heaton RK, Akshoomoff N, Tulsky D, et al. Reliability and Validity of Composite Scores from the NIH Toolbox Cognition Battery in Adults. *Journal of the International Neuropsychological Society.* 2014;20(6):588-598.
7. Wechsler D, Kodama H. *Wechsler Intelligence Scale for Children® (5th ed.).* Vol 1. 5th ed. Bloomington, MN: Pearson; 2014.
8. Bruni O, Ottaviano S, Guidetti V, et al. The Sleep Disturbance Scale for Children (SDSC) Construct ion and validation of an instrument to evaluate sleep disturbances in childhood and adolescence. *Journal of sleep research.* 1996;5(4):251-261.
9. Murtagh F, Legendre P. Ward's hierarchical agglomerative clustering method: which algorithms implement Ward's criterion? *Journal of classification.* 2014;31:274-295.
10. Troyanskaya O, Cantor M, Sherlock G, et al. Missing value estimation methods for DNA microarrays. *Bioinformatics.* 2001;17(6):520-525.
11. Su C, Zhang Y, Flory JH, et al. Clinical subphenotypes in COVID-19: derivation, validation, prediction, temporal patterns, and interaction with social determinants of health. *npj Digital Medicine.* 2021;4(1):110.

© 2023 American Medical Association. All rights reserved.

eFigure 1. An Illustration of Missing Values in the Child × SDOH Variable Matrix. According to the figure, missing values are not at random.

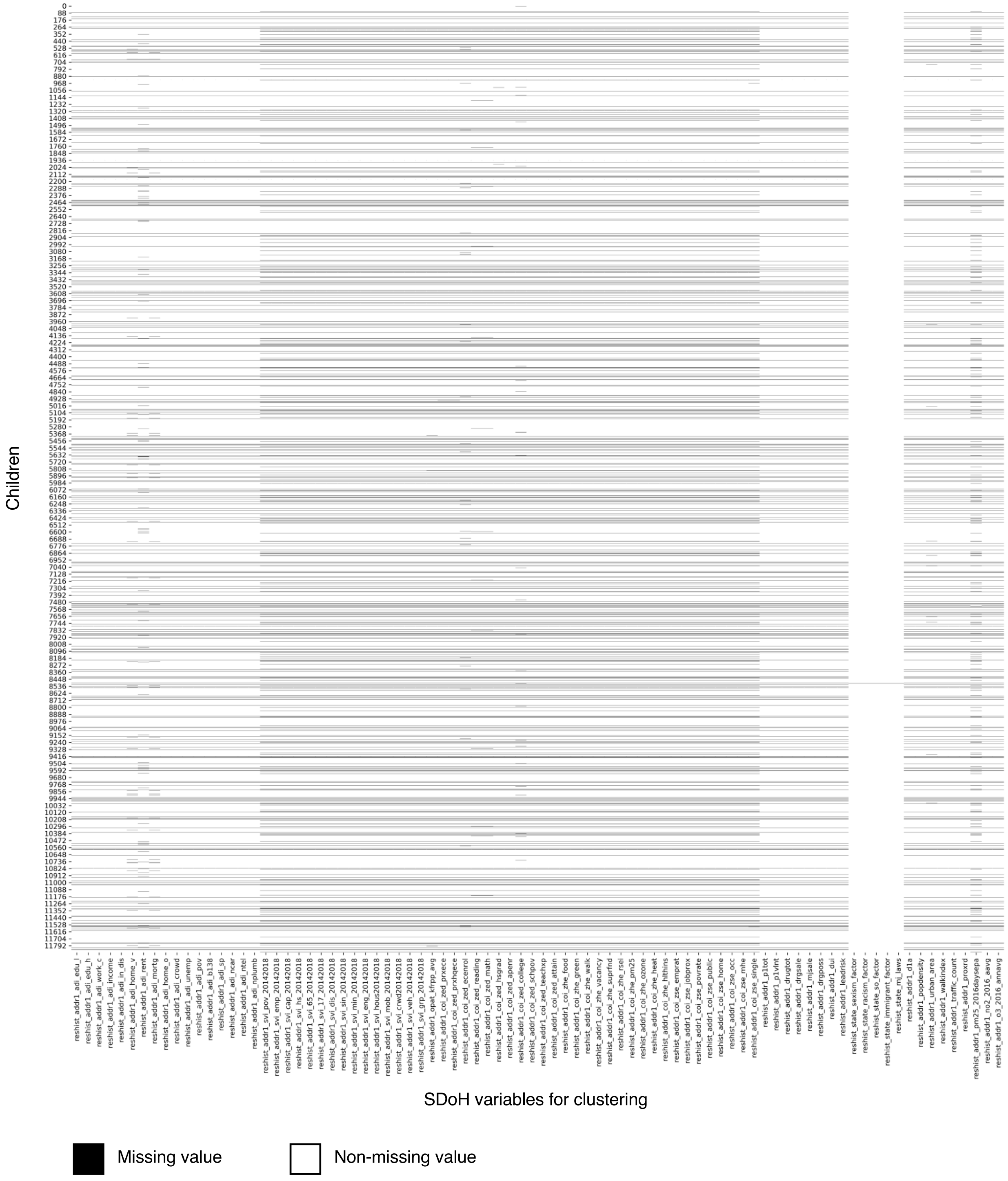

© 2023 American Medical Association. All rights reserved.

eFigure 2. Dendrogram for SDOH Pattern Identification in the Primary Analysis

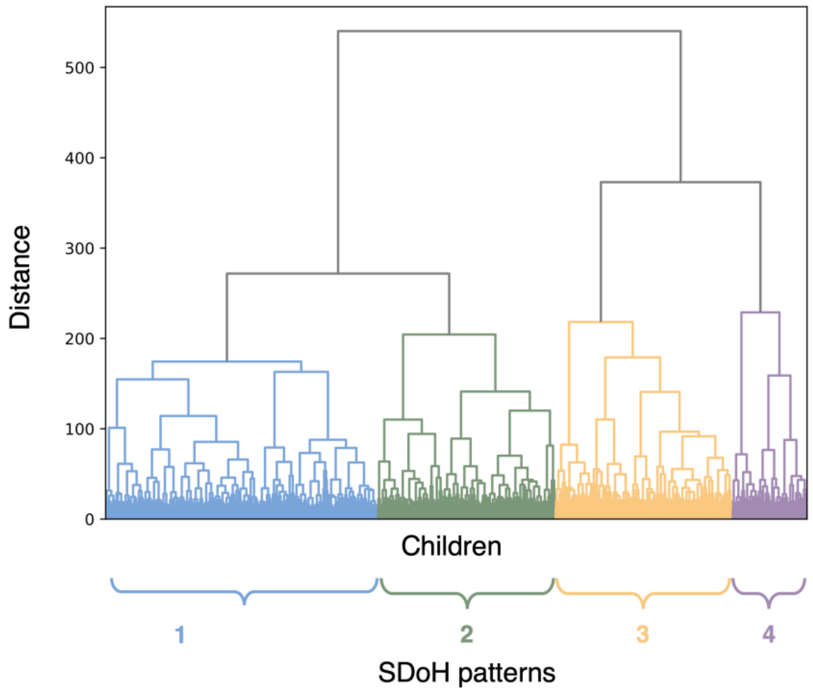

© 2023 American Medical Association. All rights reserved.

eFigure 3. Visualization of SDOH Patterns in 2D t-SNE Space

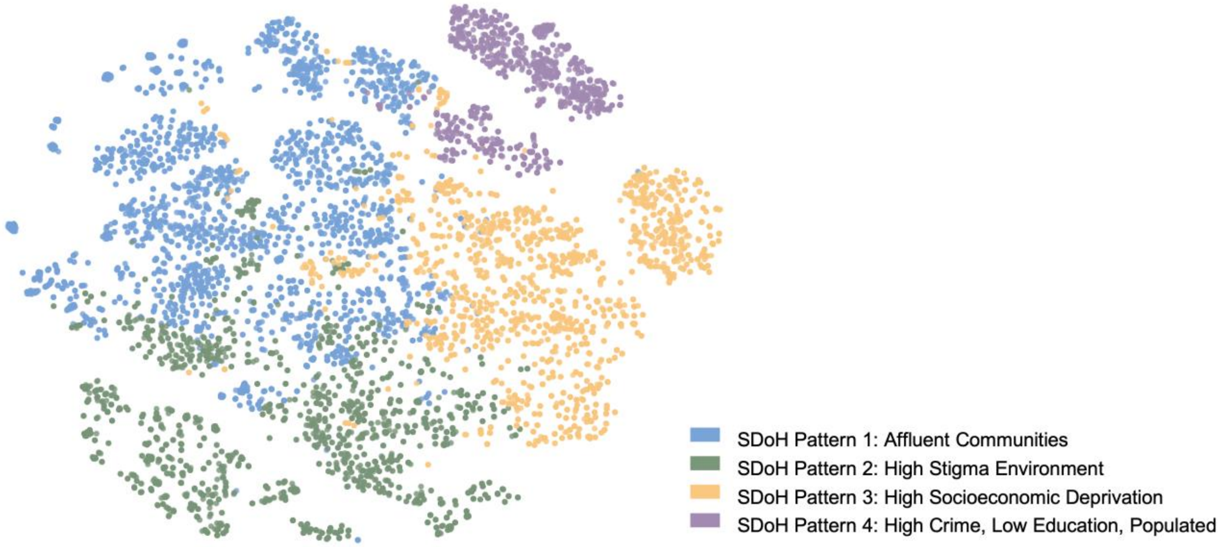

© 2023 American Medical Association. All rights reserved.

eFigure 4. Characteristics of the Identified Social Determinants of Health (SDOH) patterns (with Detailed Information)

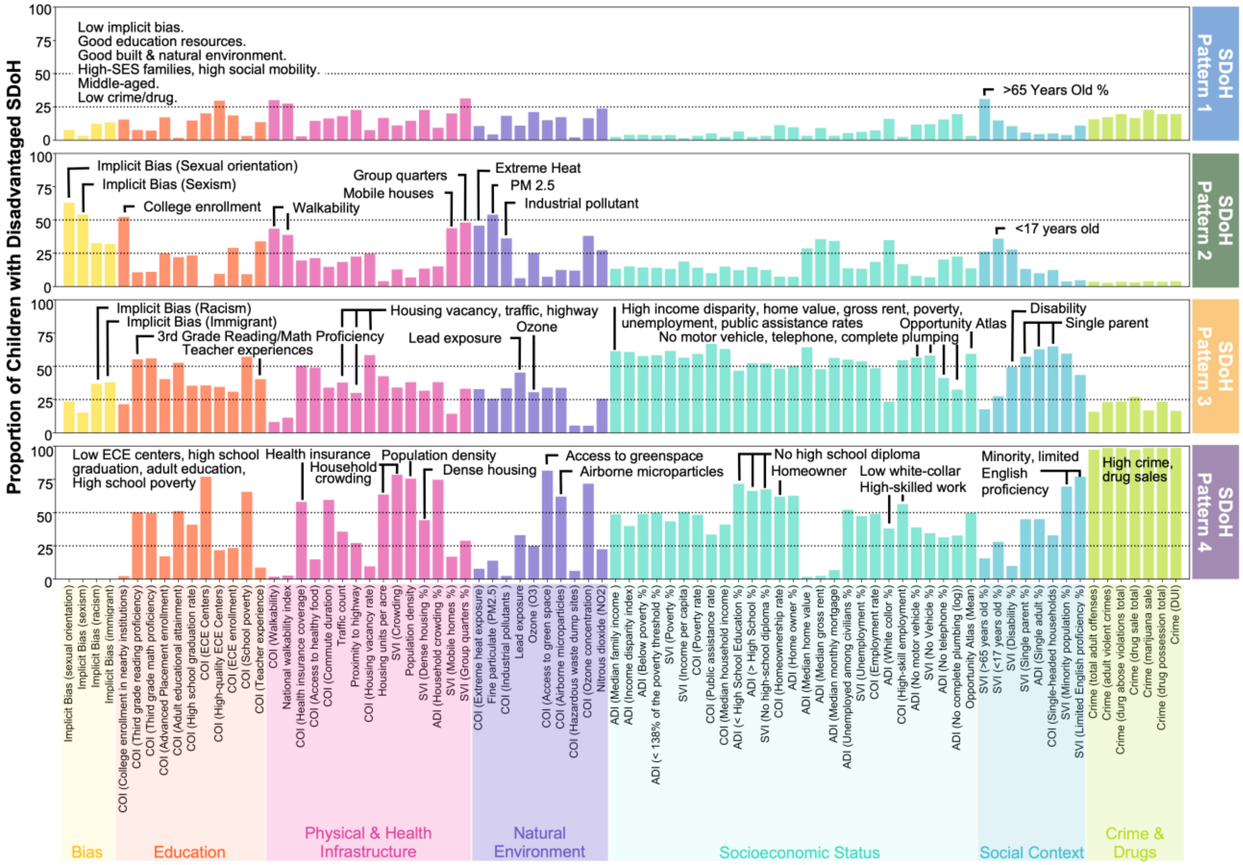

Each bar shows the proportion of children with disadvantaged status in each (continuous) SDOH variable across SDOH patterns. Children with disadvantaged SDOH are children who have worse SDOH exposures than 75% population concerning a specific SDOH variable.

Abbreviations: ADI, Area Deprivation Index; COI, Child Opportunity, Index; ECE, Early Childhood Education; SDOH, social determinants of health; SVI, Social Vulnerability Index.

© 2023 American Medical Association. All rights reserved.

eFigure 5. Association Between SDOH Patterns with Marijuana State Law Types and Census Tract Urban Classification

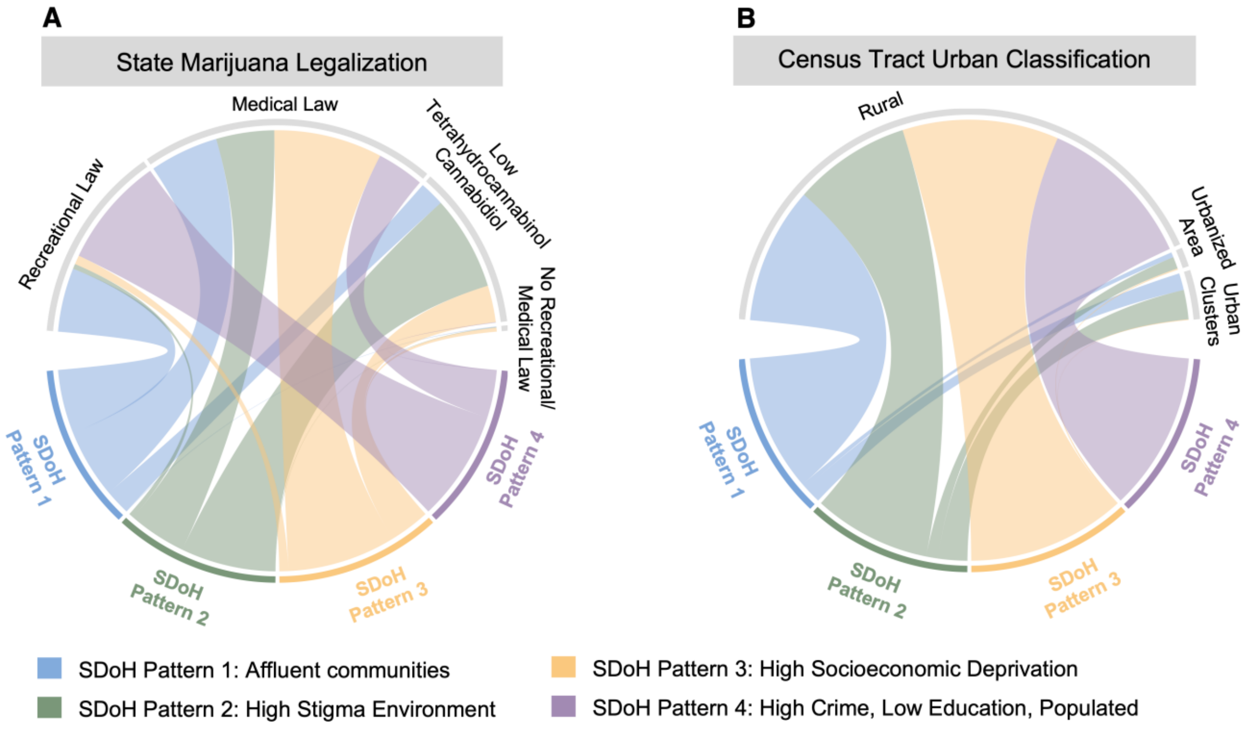

Note. (A) Chord diagram showing the association between SDOH patterns and marijuana state law types. The width of the ribbon indicates the proportion of children whose living regions are covered with a specific marijuana law type. (B) Chord diagram showing the association between SDOH patterns and census tract urban classification. The width of the ribbon indicates the proportion of children whose living regions belong to a specific census tract class.

© 2023 American Medical Association. All rights reserved.

eFigure 6. Results of Sensitivity Analysis to Dropped Samples Due to Missing Values

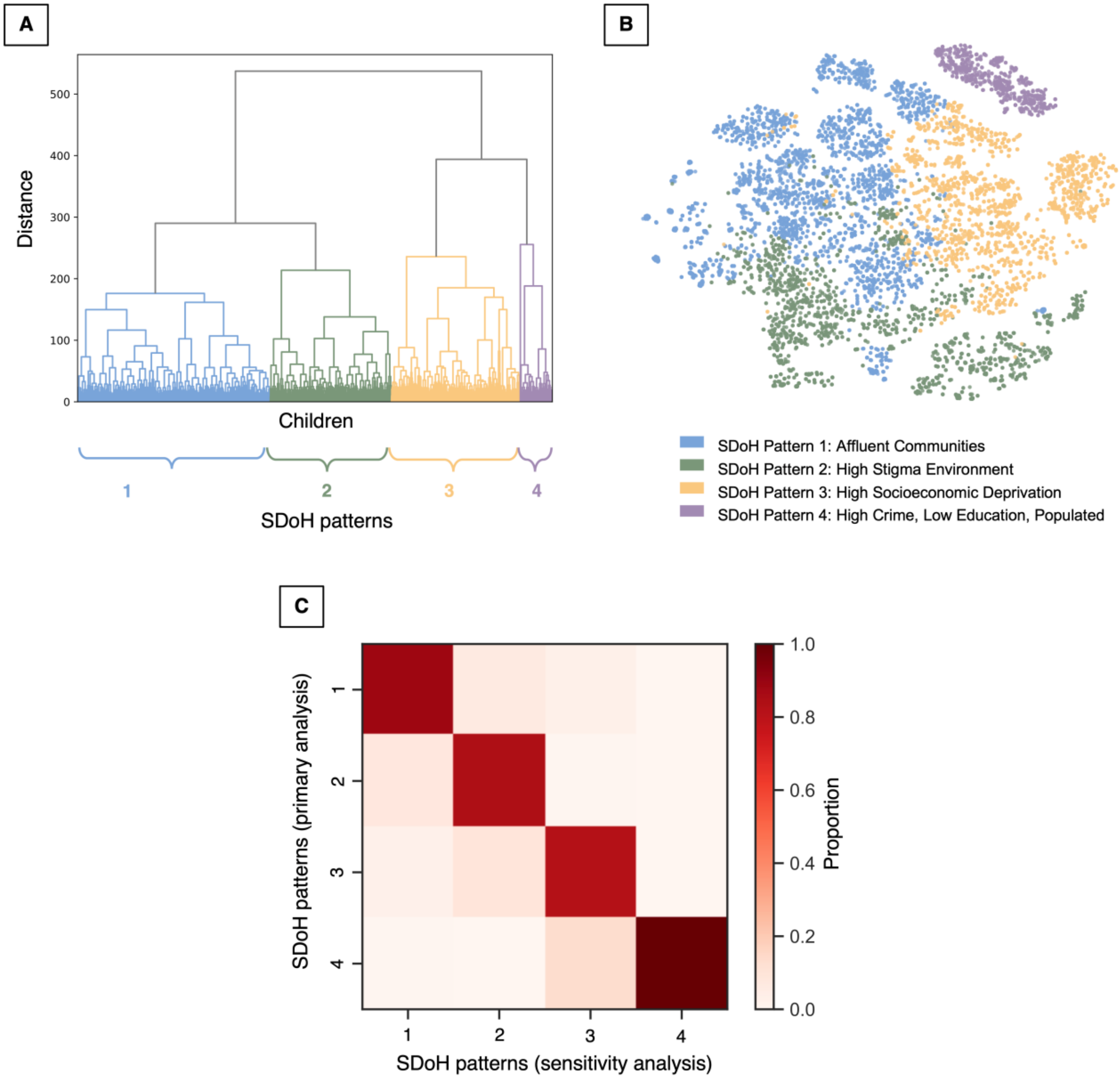

(A) Dendrogram of hierarchical clustering analysis. (B) Visualization of identified SDOH patterns in the 2D t-SNE space. (C) Confusion matrix for comparing the SDOH patterns identified by the primary analysis and sensitivity analysis. Density of color indicates the proportion of overlapped children within SDOH patterns identified by the primary analysis and sensitivity analysis.

© 2023 American Medical Association. All rights reserved.

eFigure 7. Results of Sensitivity Analysis at the Neighborhood Level

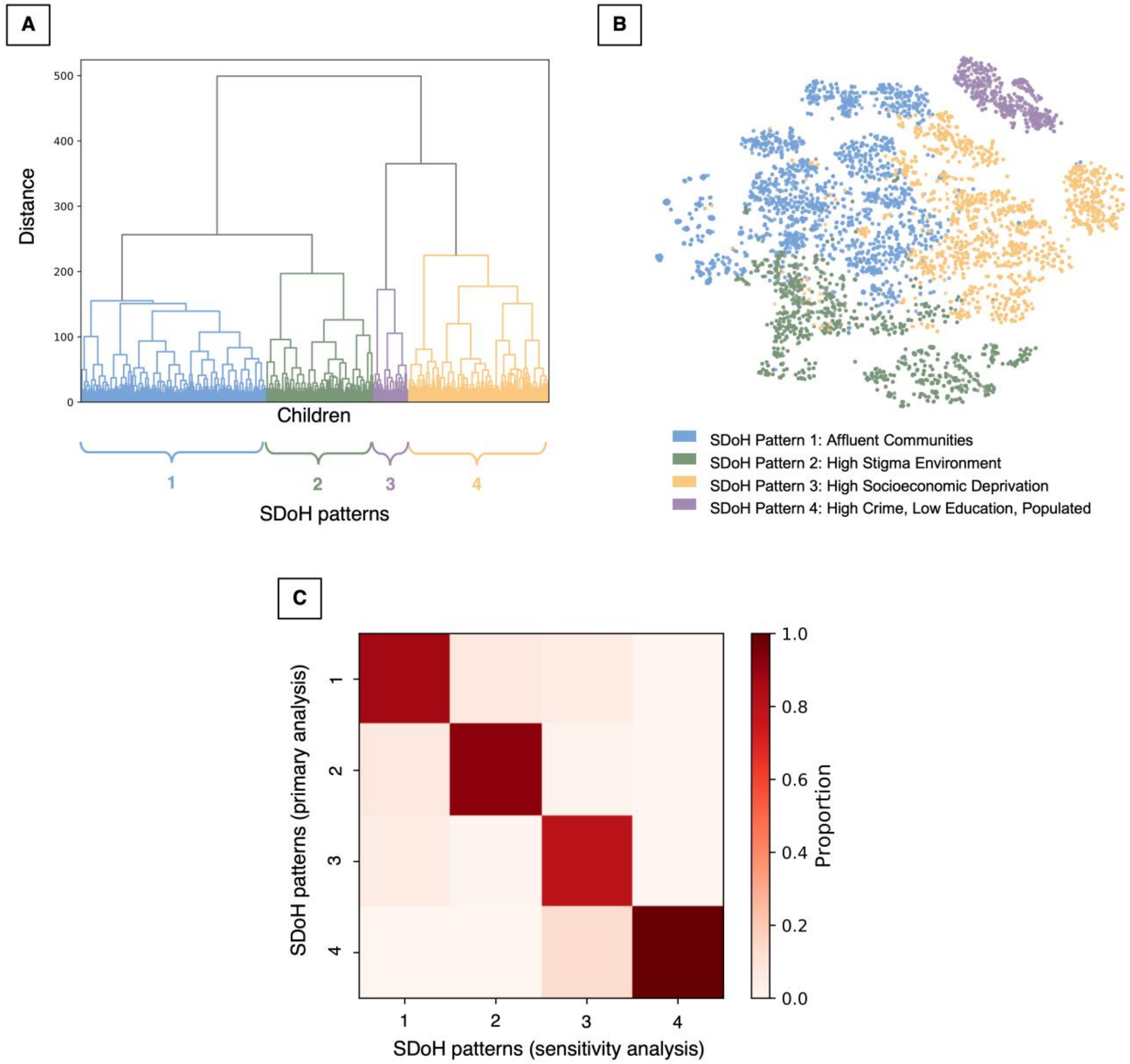

(A) Dendrogram of hierarchical clustering analysis. (B) Visualization of identified SDOH patterns in the 2D t-SNE space. (C) Confusion matrix for comparing the SDOH patterns identified by the primary analysis and sensitivity analysis. Density of color indicates the proportion of overlapped children within SDOH patterns identified by the primary analysis and sensitivity analysis.

© 2023 American Medical Association. All rights reserved.

eFigure 8. Results of Sensitivity Analysis to the Included Data Samples

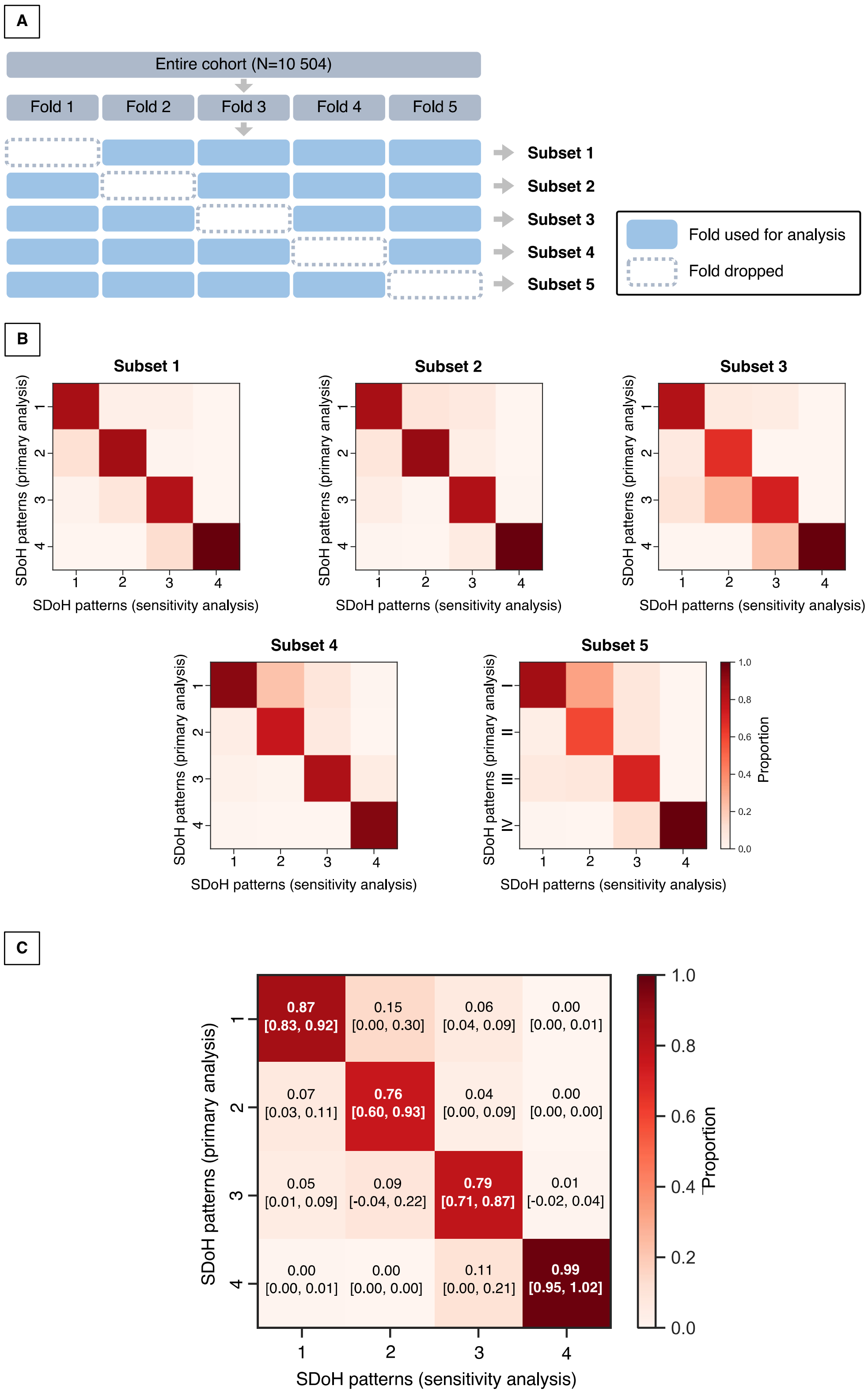

© 2023 American Medical Association. All rights reserved.

In this sensitivity analysis, we carried out the following steps: (A) we first partitioned the entire cohort into 5 folds, then each time we successive dropped 1 fold and used remaining 4 folds to construct a subset. This subset was then subjected to hierarchical clustering analysis to re-identify the SDOH patterns. (B) We created confusion matrices to compare the SDOH patterns discovered in the primary analysis with those identified in each subset during the sensitivity analysis. The depth of color in the confusion matrix signifies the proportion of children whose SDOH patterns overlapped in both the primary and sensitivity analyses. (C) We constructed an average confusion matrix by combining the results from all five subsets. Specifically, the color density of each element in this matrix corresponds to the mean value of that element across the five subsets. Alongside this, we also reported 95% confidence intervals for added precision and reliability.

© 2023 American Medical Association. All rights reserved.

eFigure 9. Forest Plot Showing Associations Between the Identified Social Determinants of Health (SDOH) Patterns and Mental Health and Suicidal Behavior Outcomes of Children at 1-Year Follow-Up

	Mental Health Outcome	SDoH Pattern 1 β [95% CI]	SDoH Pattern 2 β [95% CI]	SDoH Pattern 3 β [95% CI]	SDoH Pattern 4 β [95% CI]
**Internalizing Problems**	Anxious/Depressed	-0.12 [-0.44, 0.19]	0.22 [-0.14, 0.58]	0.08 [-0.28, 0.43]	-0.38 [-1.00, 0.24]
	Withdrawn/Depressed	-0.52 [-0.83, -0.21]	0.16 [-0.19, 0.51]	0.55 [0.21, 0.90]	-0.13 [-0.72, 0.47]
	Somatic Complaints	-0.46 [-0.77, -0.14]	0.29 [-0.07, 0.65]	0.47 [0.12, 0.83]	-0.49 [-1.09, 0.11]
	**Internalizing Problems Total Score**	-0.71 [-1.27, -0.14]	0.39 [-0.26, 1.05]	0.75 [0.12, 1.38]	-0.78 [-1.94, 0.38]
**Externalizing Problems**	Rule-Breaking Behaviors	-0.65 [-0.90, -0.40]	-0.07 [-0.36, 0.22]	0.87 [0.59, 1.14]	0.04 [-0.48, 0.56]
	Aggressive Behaviors	-0.47 [-0.75, -0.19]	-0.14 [-0.47, 0.18]	0.72 [0.41, 1.03]	0.02 [-0.56, 0.59]
	**Externalizing Problems Total Score**	-1.35 [-1.89, -0.81]	0.25 [-0.38, 0.87]	1.49 [0.89, 2.09]	-0.11 [-1.24, 1.02]
**Problem Behaviors**	Social Problems	-0.48 [-0.72, -0.24]	0.18 [-0.10, 0.45]	0.51 [0.24, 0.77]	-0.22 [-0.70, 0.25]
	Thought Problems	-0.40 [-0.71, -0.08]	0.40 [0.04, 0.77]	0.32 [-0.03, 0.68]	-0.66 [-1.29, -0.02]
	Attention Problems	-0.38 [-0.69, -0.06]	0.31 [-0.05, 0.67]	0.31 [-0.04, 0.66]	-0.39 [-1.01, 0.23]
**Mental Health Problem Total Score**		-1.23 [-1.83, -0.63]	0.64 [-0.06, 1.34]	1.15 [0.48, 1.82]	-0.78 [-2.05, 0.50]
**Suicidal Behaviors**	Suicide Attempts	-0.35 [-0.73, 0.03]	0.19 [-0.19, 0.58]	0.09 [-0.32, 0.50]	0.24 [-0.32, 0.80]
	Suicide Ideation	-0.08 [-0.23, 0.07]	0.10 [-0.06, 0.25]	0.13 [-0.04, 0.30]	-0.27 [-0.53, -0.02]

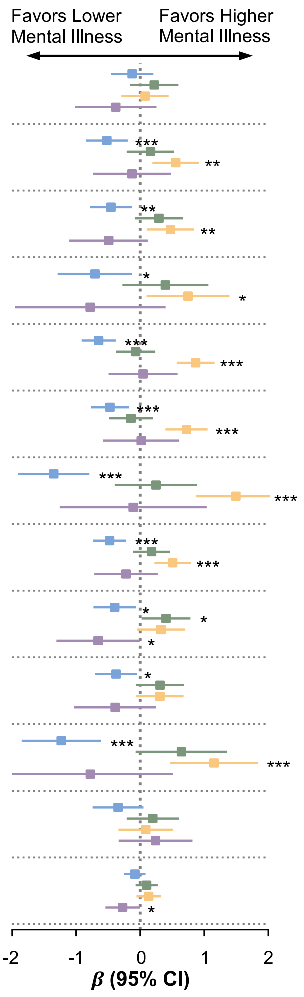

SDoH Pattern 1: Affluent communities

SDoH Pattern 2: High Structural Discrimination

SDoH Pattern 3: High Socioeconomic Deprivation

SDoH Pattern 4: High Crime, Low Education, Populated

* $P < 0.05$ ** $P < 0.01$ *** $P < 0.001$

We measured child mental health outcomes based on Child Behavior Checklist (CBCL) symptom scales (continuous) and self-report suicidal behaviors. Higher CBCL value on a CBCL symptom scale indicates worse mental illness of the child. For continuous outcomes, i.e., CBCL symptom scales, β (95% CI) and P values were estimated based on linear mixed effect regressions analyses, adjusting for baseline age, sex, and race/ethnicity and including a random-effects term to account for within-site clustering. For binary outcomes, i.e., suicidal behaviors, β (95% CI) and P values are based on were estimated based on mixed effect logistic regression analyses, adjusting for baseline age, sex, and race/ethnicity and including a random-effects term to account for within-site clustering.

Abbreviations: CBCL, Child Behavior Checklist.

© 2023 American Medical Association. All rights reserved.

eFigure 10. Forest Plot Showing Associations Between the Identified Social Determinants of health (SDOH) Patterns and Mental Health and Suicidal Behavior Outcomes of Children at 2-Year Follow-Up

		β [95% CI]			
**Mental Health Outcome**		**SDoH Pattern 1**	**SDoH Pattern 2**	**SDoH Pattern 3**	**SDoH Pattern 4**
**Internalizing Problems**	Anxious/Depressed	-0.06 [-0.40, 0.28]	0.06 [-0.33, 0.45]	0.15 [-0.24, 0.54]	-0.35 [-0.98, 0.29]
	Withdrawn/Depressed	-0.43 [-0.77, -0.09]	-0.11 [-0.49, 0.28]	0.64 [0.25, 1.03]	0.08 [-0.55, 0.71]
	Somatic Complaints	-0.65 [-1.00, -0.29]	0.45 [0.05, 0.85]	0.44 [0.03, 0.85]	-0.13 [-0.81, 0.55]
	**Internalizing Problems Total Score**	-0.52 [-1.16, 0.13]	0.03 [-0.70, 0.77]	0.78 [0.05, 1.51]	-0.48 [-1.75, 0.79]
**Externalizing Problems**	Rule-Breaking Behaviors	-0.64 [-0.89, -0.39]	0.05 [-0.23, 0.33]	0.87 [0.59, 1.15]	-0.24 [-0.70, 0.23]
	Aggressive Behaviors	-0.50 [-0.80, -0.20]	0.07 [-0.27, 0.41]	0.67 [0.33, 1.01]	-0.28 [-0.86, 0.30]
	**Externalizing Problems Total Score**	-1.44 [-2.04, -0.84]	0.41 [-0.27, 1.09]	1.50 [0.82, 2.18]	-0.13 [-1.31, 1.06]
**Problem Behaviors**	Social Problems	-0.49 [-0.78, -0.21]	0.05 [-0.28, 0.37]	0.63 [0.31, 0.96]	-0.17 [-0.72, 0.38]
	Thought Problems	-0.35 [-0.69, -0.01]	0.29 [-0.10, 0.67]	0.42 [0.03, 0.82]	-0.69 [-1.34, -0.05]
	Attention Problems	-0.64 [-0.97, -0.31]	0.38 [0.01, 0.76]	0.54 [0.16, 0.91]	-0.25 [-0.88, 0.39]
**Mental Health Problem Total Score**		-1.01 [-1.71, -0.31]	0.45 [-0.34, 1.24]	1.01 [0.22, 1.80]	-0.55 [-1.96, 0.87]
**Suicidal Behaviors**	Suicide Attempts	-0.62 [-1.02, -0.21]	0.39 [0.00, 0.78]	0.24 [-0.18, 0.66]	0.14 [-0.48, 0.76]
	Suicide Ideation	-0.12 [-0.27, 0.04]	0.08 [-0.09, 0.25]	0.07 [-0.11, 0.25]	-0.01 [-0.28, 0.26]

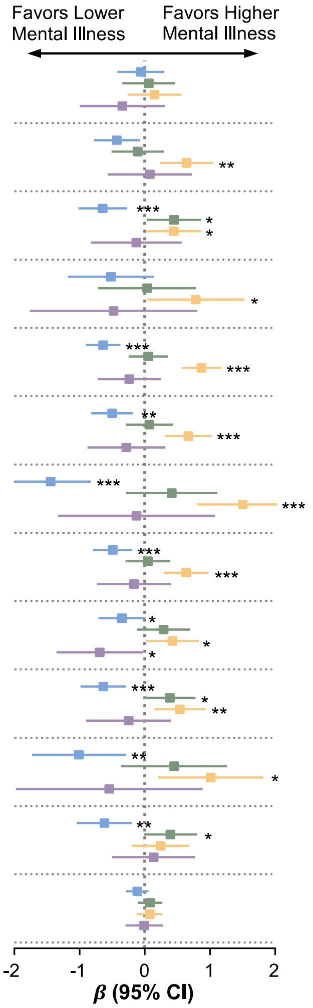

SDoH Pattern 1: Affluent communities
SDoH Pattern 2: High Structural Discrimination
SDoH Pattern 3: High Socioeconomic Deprivation
SDoH Pattern 4: High Crime, Low Education, Populated

* $P < 0.05$ ** $P < 0.01$ *** $P < 0.001$

We measured child mental health outcomes based on Child Behavior Checklist (CBCL) symptom scales (continuous) and self-report suicidal behaviors. Higher CBCL value on a CBCL symptom scale indicates worse mental illness of the child. For continuous outcomes, i.e., CBCL symptom scales, β (95% CI) and P values were estimated based on linear mixed effect regression analyses, adjusting for baseline age, sex, and race/ethnicity and including a random-effects term to account for within-site clustering. For binary outcomes, i.e., suicidal behaviors, β (95% CI) and P values are based on were estimated based on mixed effect logistic regression analyses, adjusting for baseline age, sex, and race/ethnicity and including a random-effects term to account for within-site clustering.

Abbreviations: CBCL, Child Behavior Checklist.

© 2023 American Medical Association. All rights reserved.

eFigure 11. Forest Plot Showing Associations Between the Identified Social Determinants of Health (SDOH) Patterns and Physical Health Outcomes of Children

β [95% CI]

Physical Health Outcome		SDoH Pattern 1	SDoH Pattern 2	SDoH Pattern 3	SDoH Pattern 4
**General Physical Health**	Days exercise ≥60 mins in past 7 days	0.31 [0.19, 0.42]	0.02 [-0.12, 0.15]	-0.33 [-0.46, -0.20]	-0.22 [-0.45, 0.01]
	Body Mass Index (BMI)	-0.97 [-1.19, -0.76]	-0.09 [-0.34, 0.16]	1.01 [0.77, 1.25]	1.01 [0.54, 1.47]
**Sleep Disorders Scale**	Disorders of Initiating and Maintaining Sleep	-0.25 [-0.45, -0.05]	0.22 [-0.00, 0.45]	0.20 [-0.01, 0.42]	-0.36 [-0.77, 0.05]
	Sleep Breathing disorders	-0.17 [-0.23, -0.11]	0.05 [-0.02, 0.12]	0.15 [0.09, 0.22]	0.02 [-0.09, 0.13]
	Disorder of Arousal	-0.02 [-0.06, 0.02]	0.02 [-0.03, 0.07]	0.06 [0.01, 0.11]	-0.11 [-0.19, -0.04]
	Sleep-Wake Transition Disorders	-0.02 [-0.15, 0.12]	0.07 [-0.09, 0.22]	0.05 [-0.10, 0.20]	-0.27 [-0.53, -0.01]
	Disorders of Excessive Somnolence	-0.20 [-0.32, -0.07]	0.06 [-0.08, 0.21]	0.26 [0.12, 0.40]	-0.23 [-0.48, 0.02]
	Sleep Hyperhidrosis	-0.08 [-0.13, -0.02]	0.04 [-0.03, 0.10]	0.06 [-0.00, 0.13]	-0.01 [-0.12, 0.10]
	**Sleep Disorders Scale Total Score**	-0.71 [-1.14, -0.28]	0.40 [-0.11, 0.90]	0.77 [0.29, 1.25]	-0.93 [-1.83, -0.02]

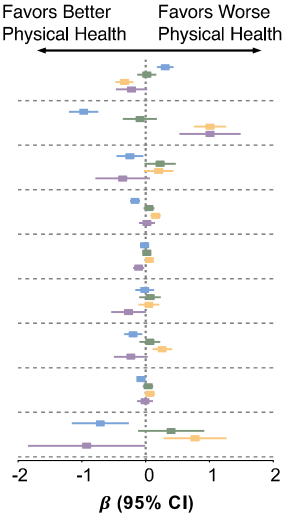

SDoH Pattern 1: Affluent communities
SDoH Pattern 2: High Stigma Environment
SDoH Pattern 3: High Socioeconomic Deprivation
SDoH Pattern 4: High Crime, Low Education, Populated

For the sleep disorder scales, a higher value indicates the worse health condition of the child, and vice versa. We measured general physical health and sleep disorder scales. For continuous outcomes, i.e., CBCL symptom scales, β (95% CI) and P values were estimated based on linear mixed effect regressions analyses, adjusting for baseline age, sex, and race/ethnicity and including a random-effects term to account for within-site clustering. For binary outcomes, i.e., suicidal behaviors, β (95% CI) and P values are based on were estimated based on mixed effect logistic regressions analyses, adjusting for baseline age, sex, and race/ethnicity and including a random-effects term to account for within-site clustering. P values can be found in eTable 3.

© 2023 American Medical Association. All rights reserved.

eTable 1. The SDOH Variables Used for SDOH Pattern Identification

Variable	Missing rate	Median [IRQ]	Description
reshist_addr1_adi_edu_l	0.05	2.49 [0.97-5.77]	Residential history derived - Area Deprivation Index: Percentage of population aged >=25 y with <9 y of education.
reshist_addr1_adi_edu_h	0.05	92.27 [84.38-96.18]	Residential history derived - Area Deprivation Index: Percentage of population aged >=25 y with at least a high school diploma.
reshist_addr1_adi_work_c	0.05	94.56 [91.57-96.71]	Residential history derived - Area Deprivation Index: Percentage of employed persons aged >=16 y in white collar occupations.
reshist_addr1_adi_income	0.05	72367.00 [49786.00-97041.25]	Residential history derived - Area Deprivation Index: Median family income
reshist_addr1_adi_in_dis	0.05	2.06 [1.26-2.96]	Residential history derived - Area Deprivation Index: Income disparity defined by Singh as the log of 100 x ratio of the number of households with <10000 annual income to the number of households with >50000 annual income.
reshist_addr1_adi_home_v	0.06	221700.00 [145300.00-315100.00]	Residential history derived - Area Deprivation Index: Median home value.
reshist_addr1_adi_rent	0.06	1042.00 [848.00-1314.00]	Residential history derived - Area Deprivation Index: Median gross rent.
reshist_addr1_adi_mortg	0.06	1381.00 [1049.00-1716.00]	Residential history derived - Area Deprivation Index: Median monthly mortgage.
reshist_addr1_adi_home_o	0.05	69.96 [50.17-83.50]	Residential history derived - Area Deprivation Index: Percentage of owner.
reshist_addr1_adi_crowd	0.05	1.65 [0.50-3.83]	Residential history derived - Area Deprivation Index: Percentage of occupied housing units with >1 person per room (crowding).
reshist_addr1_adi_unemp	0.05	7.50 [5.03-11.21]	Residential history derived - Area Deprivation Index: Percentage of civilian labor force population aged >=16 y unemployed (unemployment rate).
reshist_addr1_adi_pov	0.05	7.15 [3.19-15.78]	Residential history derived - Area Deprivation Index: Percentage of families below the poverty level.
reshist_addr1_adi_b138	0.05	16.12 [9.06-30.20]	Residential history derived - Area Deprivation Index: Percentage of population below 138% of the poverty threshold.
reshist_addr1_adi_sp	0.05	14.37 [8.92-23.46]	Residential history derived - Area Deprivation Index: Percentage of single.
reshist_addr1_adi_ncar	0.05	4.96 [2.18-10.85]	Residential history derived - Area Deprivation Index: Percentage of occupied housing units without a motor vehicle.
reshist_addr1_adi_ntel	0.05	1.65 [0.76-2.91]	Residential history derived - Area Deprivation Index: Percentage of occupied housing units without a telephone.
reshist_addr1_adi_nplumb	0.05	0.00 [0.00-0.34]	Residential history derived - Area Deprivation Index: Percentage of occupied housing units without complete plumbing (log).
reshist_addr1_svi_pov_20142018	0.08	0.37 [0.15-0.68]	Residential history derived - Census tract CDC SVI (percentile % below poverty subcomponent) at primary residential address (ACS 2014-2018 5 yr avg)
reshist_addr1_svi_emp_20142018	0.08	0.41 [0.19-0.69]	Residential history derived - Census tract CDC SVI (percentile unemployment rate subcomponent) at primary residential address (ACS 2014-2018 5 yr avg)
reshist_addr1_svi_cap_20142018	0.08	0.38 [0.18-0.67]	Residential history derived - Census tract CDC SVI (percentile per capita income subcomponent) at primary residential address (ACS 2014-2018 5 yr avg)
reshist_addr1_svi_hs_20142018	0.08	0.34 [0.14-0.64]	Residential history derived - Census tract CDC SVI (percentile % no high school diploma subcomponent) at primary residential address (ACS 2014-2018 5 yr avg)
reshist_addr1_svi_65_20142018	0.08	0.39 [0.18-0.65]	Residential history derived - Census tract CDC SVI (percentile % persons 65 and older subcomponent) at primary residential address (ACS 2014-2018 5 yr avg)
reshist_addr1_svi_17_20142018	0.08	0.56 [0.30-0.81]	Residential history derived - Census tract CDC SVI (percentile % persons 17 and younger subcomponent) at primary residential address (ACS 2014-2018 5 yr avg)
reshist_addr1_svi_dis_20142018	0.08	0.33 [0.16-0.57]	Residential history derived - Census tract CDC SVI (percentile % population with a disability subcomponent) at primary residential address (ACS 2014-2018 5 yr avg)
reshist_addr1_svi_sin_20142018	0.08	0.47 [0.24-0.73]	Residential history derived - Census tract CDC SVI (percentile % single parent households with children under 18 subcomponent) at primary residential address (ACS 2014-2018 5 yr avg)
reshist_addr1_svi_min_20142018	0.08	0.49 [0.30-0.76]	Residential history derived - Census tract CDC SVI (percentile % minority subcomponent) at primary residential address (ACS 2014-2018 5 yr avg)
reshist_addr1_svi_eng_20142018	0.08	0.50 [0.28-0.74]	Residential history derived - Census tract CDC SVI (percentile % persons who speak English "less than well" subcomponent) at primary residential address (ACS 2014-2018 5 yr avg)
reshist_addr1_svi_hous20142018	0.08	0.59 [0.32-0.78]	Residential history derived - Census tract CDC SVI (percentile % housing structures with 10 or more units subcomponent) at primary residential address (ACS 2014-2018 5 yr avg)
reshist_addr1_svi_mob_20142018	0.08	0.00 [0.00-0.57]	Residential history derived - Census tract CDC SVI (percentile % mobile homes subcomponent) at primary residential address (ACS 2014-2018 5 yr avg)
reshist_addr1_svi_crwd20142018	0.08	0.47 [0.24-0.72]	Residential history derived - Census tract CDC SVI (percentile % crowding subcomponent) at primary residential address (ACS 2014-2018 5 yr avg)
reshist_addr1_svi_veh_20142018	0.08	0.44 [0.19-0.72]	Residential history derived - Census tract CDC SVI (percentile % households with no vehicle) at primary residential address (ACS 2014-2018 5 yr avg)
reshist_addr1_svi_grp_20142018	0.08	0.44 [0.00-0.68]	Residential history derived - Census tract CDC SVI (percentile % persons in group quarters) at primary residential address (ACS 2014-2018 5 yr avg)
reshist_addr1_opat_kfrpp_avg	0.08	0.54 [0.46-0.60]	Residential history derived - Opportunity Atlas Mean outcome for all children
reshist_addr1_coi_zed_prxece	0.08	0.64 [0.10-1.01]	Educational Domain High-quality early childhood education centers: Number of ECE centers within a 5-mile radius, converted to natural log units, transformed to z-scores.
reshist_addr1_coi_zed_prxhqece	0.08	0.77 [0.58-0.88]	Educational Domain Access to healthy food: Number of NAEYC accredited centers within a 5-mile radius, converted to natural log units, transformed to z-scores.
reshist_addr1_coi_zed_ecenrol	0.09	0.22 [-0.48-0.89]	Educational Domain High school graduation rate: Percentage 3- and 4-year-olds enrolled in nursery school, preschool or kindergarten, transformed to z-scores.
reshist_addr1_coi_zed_reading	0.09	0.38 [-0.99-1.40]	Educational Domain School poverty: Percentage third graders scoring proficient on standardized reading tests, converted to NAEP scale score points, transformed to z-scores.
reshist_addr1_coi_zed_math	0.09	0.32 [-1.30-1.55]	Educational Domain Third grade reading proficiency: Percentage third graders scoring proficient on standardized math tests, converted to NAEP scale score points, transformed to z-scores.
reshist_addr1_coi_zed_hsgrad	0.09	0.36 [-0.51-0.86]	Educational Domain Third grade math proficiency: Percentage ninth graders graduating from high school on time, transformed to z-scores.
reshist_addr1_coi_zed_apenr	0.08	0.42 [-0.14-1.19]	Educational Domain Adult educational attainment: Ratio of students enrolled in at least one AP course to the number of 11th and 12th graders, transformed to z-scores.
reshist_addr1_coi_zed_college	0.09	0.42 [-0.16-1.01]	Educational Domain Early childhood education enrollment: Percentage 18-24 year-olds enrolled in college within 25-mile radius, transformed to z-scores.
reshist_addr1_coi_zed_schpov	0.08	0.21 [-0.77-1.10]	Educational Domain Teacher experience: Percentage students in elementary schools eligible for free or reduced-price lunches, transformed to z-scores and multiplied by -1.
reshist_addr1_coi_zed_teachxp	0.08	-0.15 [-0.91-0.40]	Educational Domain Early childhood education centers: Percentage teachers in their first and second year, transformed to z-scores and multiplied by -1.

© 2023 American Medical Association. All rights reserved.

reshist_addr1_coi_zed_attain	0.08	0.48 [-0.37-1.50]	Educational Domain College enrollment in nearby institutions: Percentage adults ages 25 and over with a college degree or higher, transformed to z-scores.
reshist_addr1_coi_zhe_food	0.08	0.37 [-0.20-0.68]	Health and Environment Domain Access to green space: Percentage households without a car located further than a half-mile from the nearest supermarket, transformed to z-scores and multiplied by -1.
reshist_addr1_coi_zhe_green	0.08	-0.33 [-0.95-0.55]	Health and Environment Domain Extreme heat exposure: Percentage impenetrable surface areas such as rooftops, roads or parking lots, transformed to z-scores and multiplied by -1.
reshist_addr1_coi_zhe_walk	0.08	0.47 [-0.43-1.37]	Health and Environment Domain Hazardous waste dump sites: EPA Walkability Index, transformed to z-scores.
reshist_addr1_coi_zhe_vacancy	0.08	0.52 [-0.08-0.95]	Health and Environment Domain Walkability: Percentage housing units that are vacant, transformed to z-scores and multiplied by -1.
reshist_addr1_coi_zhe_suprfnd	0.08	0.27 [0.27-0.27]	Health and Environment Domain Industrial pollutants in air, water or soil: Average number of Superfund sites within a 2-mile radius, converted to natural log units, transformed to z-scores and multiplied by -1.
reshist_addr1_coi_zhe_rsei	0.08	-0.40 [-0.71-0.49]	Industrial pollutants in air, water or soil: Index of toxic chemicals released by industrial facilities, converted to natural log units, transformed to z-scores and multiplied by -1.
reshist_addr1_coi_zhe_pm25	0.08	0.51 [-0.43-0.99]	Health and Environment Domain Housing vacancy rate: Mean estimated microparticle (PM2.5) concentration, transformed to z-scores and multiplied by -1.
reshist_addr1_coi_zhe_ozone	0.08	0.64 [0.05-1.06]	Health and Environment Domain Airborne microparticles: Mean estimated 8-hour average ozone concentration, transformed to z-scores and multiplied by -1.
reshist_addr1_coi_zhe_heat	0.08	0.71 [0.27-0.93]	Health and Environment Domain Health insurance coverage: Summer days with maximum temperature above 90F, transformed to z-scores and multiplied by -1.
reshist_addr1_coi_zhe_hlthins	0.08	0.86 [0.29-1.21]	Health and Environment Domain Ozone concentration: Percentage individuals ages 0-64 with health insurance coverage, transformed to z-scores.
reshist_addr1_coi_zse_emprat	0.08	0.57 [-0.00-1.03]	Employment rate: Percentage adults ages 25-54 who are employed, transformed to z-scores.
reshist_addr1_coi_zse_jobprox	0.08	0.42 [-0.18-0.78]	Commute duration: Percentage workers commuting more than one hour one way, transformed to z-scores and multiplied by -1.
reshist_addr1_coi_zse_povrate	0.08	0.48 [-0.26-0.89]	Social and Economic Domain: Poverty rate z-score
reshist_addr1_coi_zse_public	0.08	0.42 [-0.43-0.84]	Social and Economic Domain: Public assistance rate z-score
reshist_addr1_coi_zse_home	0.08	0.07 [-0.85-0.73]	Social and Economic Domain: Homeownership rate z-score
reshist_addr1_coi_zse_occ	0.08	0.50 [-0.35-1.37]	Social and Economic Domain: High-skill employment z-score
reshist_addr1_coi_zse_mhe	0.08	0.08 [-0.53-0.91]	Social and Economic Domain: Median household income z-score
reshist_addr1_coi_zse_single	0.08	0.27 [-0.55-0.90]	Single-headed households: Percentage family households that are single-parent headed, transformed to z-scores and multiplied by -1.
reshist_addr1_p1tot	0.05	5240.33 [1460.00-11593.33]	Residential history derived - Uniform Crime Reports: total adult offenses 1
reshist_addr1_p1vlnt	0.05	1084.00 [200.67-1947.67]	Residential history derived - Uniform Crime Reports: adult violent crimes 1
reshist_addr1_drugtot	0.05	2693.67 [840.67-6573.67]	Residential history derived - Uniform Crime Reports: durg abose violations total 1
reshist_addr1_drgsale	0.05	493.00 [106.00-1095.67]	Residential history derived - Uniform Crime Reports: drug sale total 1
reshist_addr1_mjsale	0.05	118.00 [50.33-437.33]	Residential history derived - Uniform Crime Reports: Marijuana sale 1
reshist_addr1_drgposs	0.05	1993.00 [590.33-5314.67]	Residential history derived - Uniform Crime Reports: drug possession total 1
reshist_addr1_dui	0.05	2265.00 [751.33-4499.00]	Residential history derived - Uniform Crime Reports: DUI 1
reshist_addr1_leadrisk	0.05	5.00 [2.00-8.00]	Estimated lead risk in census tract of primary residential address (1-10 scale)
reshist_state_sexism_factor	0.00	-0.37 [-0.93-0.61]	State level indicators of sexism from survey and implicit bias measures
reshist_state_racism_factor	0.00	-0.31 [-0.44-0.49]	State level indicators of racism from survey and implicit bias measures and state level structural variables
reshist_state_so_factor	0.00	-0.16 [-1.17-0.32]	State level indicators of bias against sexual orientation from structural variables
reshist_state_immigrant_factor	0.00	-0.45 [-0.62--0.16]	State level indicators of immigrant bias from survey and implicit bias measures and state level structural variables
reshist_state_mj_laws	0.00	- (categorical variable)	Marijuana state law during the same year as the assessment
reshist_addr1_d1a	0.05	2.64 [1.00-4.85]	Residential history derived - gross residential density 1
reshist_addr1_popdensity	0.05	1666.72 [803.61-2805.40]	Residential history derived - UN adjusted population density 1
reshist_addr1_urban_area	0.05	- (categorical variable)	Census Tract Urban Classification at current address #1
reshist_addr1_walkindex	0.05	10.67 [7.17-14.17]	Residential history derived - national walkability index 1
reshist_addr1_traffic_count	0.05	9546.69 [5404.09-15977.38]	Average Annual Daily Traffic Counts at current address #1
reshist_addr1_proxrd	0.05	826.30 [384.02-1556.07]	Residential history derived - proximity to major roads, in meters 1
reshist_addr1_pm25_2016daysepa	0.08	0.00 [0.00-1.00]	Residential history derived - number of days in 2016 PM2.5 was above EPA daily standards (35) at primary residential address at 1x1km2
reshist_addr1_no2_2016_aavg	0.05	18.82 [14.76-22.20]	Residential history derived - annual average of NO2 in 2016 at primary residential address at 1x1km2
reshist_addr1_o3_2016_annavg	0.05	40.59 [38.35-45.45]	Residential history derived - annual average of O3 in 2016 at primary residential address at 1x1km2

© 2023 American Medical Association. All rights reserved.

eTable 2. Characteristics in SDOH Variables by the Identified SDOH Patterns

SDOH sub-domain	SDOH characteristics	All	SDOH Patterns					
			SDOH Pattern 1: Affluent Communities	SDOH Pattern 2: High Stigma Environment	SDOH Pattern 3: High Socioeconomic Deprivation	SDOH Pattern 4: High Crime, Low Education, Populated	*P* value[a]	*P* value (Bonferroni correction)
		n = 10 504	n = 4078	n = 2661	n = 2653	n = 1112	-	-
Bias	State level indicators of bias against sexual orientation from structural variables, Median (IQR)	-0.16 (-1.17, 0.32)	-0.79 (-1.38, -0.37)	1.04 (-0.13, 1.04)	-0.13 (-0.37, 0.32)	-1.40 (-1.40, -1.40)	<0.001	<0.001
	State level indicators of sexism from survey and implicit bias measures, Median (IQR)	-0.37 (-0.93, 0.61)	-0.93 (-1.20, -0.57)	0.96 (0.41, 1.28)	-0.05 (-0.57, 0.61)	-0.83 (-0.83, -0.83)	<0.001	<0.001
	State level indicators of racism from survey and implicit bias measures and state level structural variables, Median (IQR)	-0.31 (-0.44, 0.49)	-0.44 (-0.96, -0.31)	0.20 (-0.29, 0.70)	0.40 (-0.33, 0.70)	-0.31 (-0.31, -0.31)	<0.001	<0.001
	State level indicators of immigrant bias from survey and implicit bias measures and state level structural variables, Median (IQR)	-0.45 (-0.62, -0.16)	-0.45 (-0.76, -0.25)	-0.26 (-0.45, 0.13)	-0.28 (-0.53, 0.32)	-1.75 (-1.75, -1.75)	<0.001	<0.001
Education	College enrollment in nearby institutions, Median (IQR)	0.41 (-0.16, 1.00)	0.57 (0.05, 1.20)	-0.20 (-0.79, 0.60)	0.41 (-0.05, 1.02)	0.73 (0.48, 0.90)	<0.001	<0.001
	Third grade reading proficiency, Median (IQR)	0.37 (-0.98, 1.35)	1.09 (0.24, 2.24)	0.58 (-0.14, 1.19)	-1.22 (-2.37, -0.03)	-1.02 (-1.88, 0.54)	<0.001	<0.001
	Third grade math proficiency, Median (IQR)	0.32 (-1.28, 1.51)	1.18 (0.20, 2.57)	0.47 (-0.36, 1.25)	-1.74 (-3.07, -0.03)	-1.24 (-2.33, 0.39)	<0.001	<0.001
	Advanced Placement (AP) course enrollment, Median (IQR)	0.42 (-0.14, 1.17)	0.74 (0.13, 1.36)	0.22 (-0.14, 0.98)	0.05 (-0.53, 0.86)	0.55 (0.11, 1.02)	<0.001	<0.001
	Adult educational attainment, Median (IQR)	0.47 (-0.37, 1.47)	1.41 (0.74, 2.16)	0.29 (-0.30, 0.95)	-0.40 (-0.78, 0.16)	-0.39 (-1.02, 0.74)	<0.001	<0.001
	High school graduation rate, Median (IQR)	0.36 (-0.52, 0.86)	0.72 (-0.05, 1.11)	0.34 (-0.44, 0.77)	-0.09 (-0.85, 0.60)	-0.17 (-0.82, 0.47)	<0.001	<0.001
	ECE Centers, Median (IQR)	0.64 (0.10, 1.01)	0.61 (0.12, 0.92)	-0.06 (-0.64, 0.33)	0.86 (0.67, 1.12)	1.17 (1.03, 1.36)	<0.001	<0.001
	High-quality ECE Centers, Median (IQR)	0.77 (0.57, 0.88)	0.79 (0.64, 0.89)	0.53 (-1.42, 0.69)	0.83 (0.73, 0.91)	0.81 (0.74, 0.87)	<0.001	<0.001
	ECE enrollment, Median (IQR)	0.22 (-0.48, 0.89)	0.53 (-0.20, 1.10)	-0.02 (-0.56, 0.56)	-0.00 (-0.66, 0.76)	0.15 (-0.46, 1.01)	<0.001	<0.001
	School poverty, Median (IQR)	0.20 (-0.78, 1.08)	1.02 (0.35, 1.42)	0.42 (-0.15, 0.92)	-0.92 (-1.45, -0.46)	-1.17 (-1.49, -0.24)	<0.001	<0.001
	Teacher experience, Median (IQR)	-0.15 (-0.89, 0.40)	0.07 (-0.45, 0.53)	-0.42 (-1.13, 0.20)	-0.56 (-1.54, 0.35)	-0.02 (-0.34, 0.34)	<0.001	<0.001
Physical & Health Infrastructure	Walkability, Median (IQR)	0.47 (-0.42, 1.37)	0.14 (-0.54, 1.18)	-0.21 (-0.81, 0.78)	1.05 (0.05, 1.52)	1.37 (1.07, 1.68)	<0.001	<0.001
	Composite index ranking census block groups according to their walkability., Median (IQR)	10.67 (7.17, 14.00)	9.33 (6.83, 13.50)	8.00 (5.83, 12.00)	12.33 (9.17, 14.67)	13.83 (12.33, 15.50)	<0.001	<0.001
	Health insurance coverage, Median (IQR)	0.86 (0.28, 1.21)	1.21 (0.97, 1.39)	0.80 (0.44, 1.07)	0.27 (-0.41, 0.79)	0.04 (-0.48, 0.67)	<0.001	<0.001
	Access to healthy food, Median (IQR)	0.37 (-0.21, 0.66)	0.46 (0.04, 0.68)	0.34 (-0.09, 0.62)	-0.17 (-1.48, 0.53)	0.63 (0.12, 0.85)	<0.001	<0.001
	Commute duration, Median (IQR)	0.43 (-0.16, 0.78)	0.51 (0.07, 0.81)	0.62 (0.24, 0.87)	0.28 (-0.55, 0.73)	-0.34 (-0.89, 0.17)	<0.001	<0.001
	Traffic counts modeled at the 1 km2 resolution, Median (IQR)	9613.64 (5413.19, 16086.32)	8173.06 (4540.07, 13433.80)	8148.87 (4362.60, 13916.35)	12502.90 (7729.38, 22861.87)	12715.43 (8000.42, 19694.09)	<0.001	<0.001
	Number of meters away from major road or highway., Median (IQR)	832.49 (384.82, 1557.32)	904.98 (421.21, 1688.04)	906.62 (421.75, 1721.10)	676.27 (322.09, 1259.48)	804.01 (363.55, 1530.85)	<0.001	<0.001
	Housing vacancy rate, Median (IQR)	0.52 (-0.09, 0.94)	0.79 (0.44, 1.10)	0.45 (-0.08, 0.85)	-0.32 (-1.24, 0.38)	0.69 (0.28, 1.05)	<0.001	<0.001
	Housing units per acre from EPA's Smart Location Database, Median (IQR)	2.64 (1.03, 4.81)	2.11 (0.70, 3.85)	1.31 (0.32, 2.59)	4.14 (2.52, 6.97)	5.74 (3.83, 8.70)	<0.001	<0.001
	Crowding*, Median (IQR)	0.47 (0.24, 0.72)	0.34 (0.00, 0.56)	0.43 (0.24, 0.63)	0.63 (0.36, 0.79)	0.95 (0.76, 0.98)	<0.001	<0.001
	Population Count Adjusted to Match 2015 Revision of UN WPP Country Totals in persons per 1 km2., Median (IQR)	1674.87 (815.09, 2798.78)	1365.80 (659.02, 2251.04)	993.86 (374.96, 1791.39)	2310.78 (1506.47, 3531.40)	4329.56 (2851.92, 6248.66)	<0.001	<0.001

© 2023 American Medical Association. All rights reserved.

	Percentage of housing in structures with 10+ units, Median (IQR)	0.59 (0.33, 0.78)	0.59 (0.33, 0.77)	0.45 (0.22, 0.66)	0.64 (0.42, 0.82)	0.74 (0.53, 0.89)	<0.001	<0.001
	Percentage of occupied housing units with >1 person per room (crowding), Median (IQR)	1.67 (0.50, 3.90)	0.78 (0.00, 2.01)	1.42 (0.54, 2.79)	2.76 (1.07, 5.24)	13.06 (3.65, 24.67)	<0.001	<0.001
	Percentage of mobile homes, Median (IQR)	0.00 (0.00, 0.58)	0.00 (0.00, 0.52)	0.51 (0.00, 0.75)	0.00 (0.00, 0.49)	0.00 (0.00, 0.49)	<0.001	<0.001
	Census Tract Urban Classification at current address, N (%)							
	Rural	9213 (87.71)	3512 (86.12)	1972 (74.11)	2617 (98.64)	1112 (100.00)	<0.001	<0.001
	Urban Clusters	352 (3.35)	131 (3.21)	198 (7.44)	23 (0.87)	0 (0.00)		
	Urbanized Area	939 (8.94)	435 (10.67)	491 (18.45)	13 (0.49)	0 (0.00)		
	Percentage of persons living in group quarters , Median (IQR)	0.44 (0.00, 0.68)	0.44 (0.00, 0.68)	0.38 (0.00, 0.61)	0.50 (0.00, 0.71)	0.50 (0.00, 0.70)	<0.001	<0.001
Natural Environment	Extreme heat exposure, Median (IQR)	0.70 (0.27, 0.92)	0.80 (0.57, 0.98)	0.27 (-0.69, 0.74)	0.67 (-0.08, 0.81)	0.81 (0.56, 1.05)	<0.001	<0.001
	Fine particulate (PM2.5), spatio-temporal model predictions measured in μg/m3 at 1 km2 resolution, Median (IQR)	0.00 (0.00, 1.00)	0.00 (0.00, 0.00)	3.00 (0.00, 6.00)	0.00 (0.00, 2.00)	0.00 (0.00, 0.00)	<0.001	<0.001
	Industrial pollutants in air, water or soil, Median (IQR)	-0.40 (-0.72, 0.50)	-0.32 (-0.63, 0.71)	-0.46 (-0.85, 0.05)	-0.59 (-0.83, 0.27)	0.13 (-0.34, 0.55)	<0.001	<0.001
	Estimated lead risk in census tract of primary residential address (1-10 scale), Median (IQR)	5.00 (2.00, 8.00)	3.00 (2.00, 6.00)	3.00 (2.00, 5.00)	8.00 (5.00, 10.00)	7.00 (5.00, 9.00)	<0.001	<0.001
	Ozone (O3), spatio-temporal model predictions measured in predictions measured in μg/m3 at 1km2 resolution, Median (IQR)	40.57 (38.31, 45.42)	40.80 (38.52, 44.87)	40.04 (37.26, 45.46)	40.15 (38.70, 46.46)	42.37 (38.92, 45.42)	<0.001	<0.001
	Access to green space, Median (IQR)	-0.34 (-0.94, 0.53)	-0.04 (-0.61, 0.74)	0.42 (-0.52, 0.98)	-0.68 (-1.15, -0.26)	-1.50 (-1.89, -1.08)	<0.001	<0.001
	Airborne microparticles, Median (IQR)	0.52 (-0.43, 0.99)	0.73 (-0.28, 1.15)	0.74 (-0.04, 0.99)	-0.05 (-0.64, 0.87)	-0.80 (-1.87, -0.34)	<0.001	<0.001
	Hazardous waste dump sites, Median (IQR)	0.27 (0.27, 0.27)	0.27 (0.27, 0.27)	0.27 (0.27, 0.27)	0.27 (0.27, 0.27)	0.27 (0.27, 0.27)	<0.001	<0.001
	Ozone concentration, Median (IQR)	0.63 (0.04, 1.06)	0.75 (0.45, 1.55)	0.22 (-0.42, 0.85)	0.78 (0.51, 1.23)	-0.24 (-1.42, 0.08)	<0.001	<0.001
	Nitrous dioxide (NO2), spatio-temporal model predictions measured in ppb (parts per billion) at 1 km2 resolution, Median (IQR)	18.74 (14.75, 22.19)	18.75 (14.11, 21.95)	15.93 (11.93, 23.30)	19.33 (16.18, 22.26)	19.78 (18.32, 21.85)	<0.001	<0.001
Socioeconomic Status	Median family income, Median (IQR)	72484.00 (50371.25, 96354.00)	96600.00 (78635.00, 118175.75)	70804.00 (60326.00, 84839.00)	45250.00 (34395.00, 57667.00)	51955.00 (36020.00, 76858.00)	<0.001	<0.001
	Income disparity defined by Singh (2003), Median (IQR)	2.05 (1.28, 2.93)	1.40 (0.71, 2.00)	1.92 (1.30, 2.59)	3.27 (2.53, 4.01)	2.64 (1.95, 3.39)	<0.001	<0.001
	Percentage of families below the poverty level, Median (IQR)	7.14 (3.27, 15.53)	3.66 (1.70, 6.54)	6.58 (3.72, 11.19)	18.17 (10.33, 28.19)	14.84 (7.17, 27.86)	<0.001	<0.001
	Percentage of population below 138% of the poverty threshold, Median (IQR)	16.04 (9.13, 29.57)	9.56 (5.70, 14.54)	15.16 (9.82, 21.90)	33.00 (23.07, 44.37)	29.74 (16.87, 46.07)	<0.001	<0.001
	Percentage of persons living in poverty*, Median (IQR)	0.37 (0.15, 0.67)	0.18 (0.09, 0.36)	0.32 (0.15, 0.50)	0.75 (0.57, 0.89)	0.63 (0.36, 0.82)	<0.001	<0.001
	Per capita income, Median (IQR)	0.38 (0.18, 0.67)	0.18 (0.09, 0.28)	0.43 (0.29, 0.62)	0.73 (0.54, 0.88)	0.68 (0.27, 0.91)	<0.001	<0.001
	Poverty rate, Median (IQR)	0.48 (-0.25, 0.89)	0.85 (0.51, 1.01)	0.59 (0.20, 0.85)	-0.47 (-1.29, 0.09)	-0.20 (-0.97, 0.49)	<0.001	<0.001
	Public assistance rate, Median (IQR)	0.41 (-0.43, 0.83)	0.77 (0.41, 0.97)	0.53 (0.12, 0.80)	-0.95 (-2.14, -0.19)	0.05 (-0.69, 0.70)	<0.001	<0.001
	Median household income, Median (IQR)	0.08 (-0.53, 0.87)	0.83 (0.20, 1.50)	0.08 (-0.29, 0.60)	-0.71 (-1.04, -0.36)	-0.26 (-0.79, 0.38)	<0.001	<0.001
	Percentage of population aged >=25 years with <9 years of education, Median (IQR)	2.49 (0.98, 5.78)	1.39 (0.63, 2.73)	1.76 (0.83, 3.58)	5.44 (2.95, 9.00)	14.53 (4.71, 25.83)	<0.001	<0.001
	Percentage of population aged >=25 years with at least a high school diploma, Median (IQR)	92.27 (84.49, 96.14)	95.76 (92.74, 97.50)	93.21 (88.72, 95.71)	84.00 (77.01, 89.49)	75.16 (56.84, 90.21)	<0.001	<0.001
	Percentage of persons at least 25 years old without a high-school diploma, Median (IQR)	0.34 (0.14, 0.64)	0.17 (0.07, 0.32)	0.28 (0.15, 0.49)	0.65 (0.46, 0.82)	0.87 (0.47, 0.97)	<0.001	<0.001
	Homeownership rate, Median (IQR)	0.07 (-0.81, 0.72)	0.44 (-0.22, 0.93)	0.54 (-0.02, 0.87)	-0.76 (-1.48, -0.20)	-1.25 (-1.91, -0.22)	<0.001	<0.001

© 2023 American Medical Association. All rights reserved.

	Percentage of owner, Median (IQR)	70.20 (51.36, 83.26)	78.63 (64.12, 87.66)	77.91 (66.78, 84.56)	50.81 (37.24, 64.74)	42.14 (28.01, 63.63)	<0.001	<0.001
	Median home value, Median (IQR)	221150.00 (145200.00, 311725.00)	283000.00 (223700.00, 384450.00)	184900.00 (139300.00, 235300.00)	113300.00 (77700.00, 171200.00)	330300.00 (266500.00, 506600.00)	<0.001	<0.001
	Median gross rent, Median (IQR)	1044.00 (852.00, 1316.00)	1189.00 (977.00, 1555.00)	936.00 (784.00, 1121.00)	868.00 (740.00, 1090.00)	1174.50 (1033.00, 1437.25)	<0.001	<0.001
	Median monthly mortgage, Median (IQR)	1377.00 (1049.00, 1704.00)	1611.00 (1408.00, 2011.00)	1169.00 (944.00, 1389.00)	996.00 (761.00, 1315.00)	1706.00 (1367.75, 2208.50)	<0.001	<0.001
	Percentage of civilian labor force population aged >=16 y unemployed (unemployment rate), Median (IQR)	7.50 (5.06, 11.17)	5.80 (4.22, 7.86)	6.55 (4.63, 9.38)	11.77 (8.26, 16.72)	11.40 (8.37, 15.05)	<0.001	<0.001
	Unemployment rate, Median (IQR)	0.41 (0.19, 0.69)	0.27 (0.13, 0.45)	0.30 (0.15, 0.53)	0.71 (0.47, 0.88)	0.66 (0.47, 0.84)	<0.001	<0.001
	Employment rate, Median (IQR)	0.57 (0.00, 1.03)	0.94 (0.54, 1.24)	0.57 (0.15, 0.95)	0.05 (-0.76, 0.59)	0.02 (-0.47, 0.50)	<0.001	<0.001
	Percentage of employed persons aged >=16 years in white collar occupations, Median (IQR)	94.50 (91.57, 96.65)	95.33 (93.08, 97.12)	93.27 (90.60, 95.34)	94.63 (91.82, 96.95)	92.74 (90.26, 96.08)	<0.001	<0.001
	High-skill employment, Median (IQR)	0.49 (-0.35, 1.35)	1.32 (0.70, 1.91)	0.34 (-0.17, 0.93)	-0.44 (-0.91, 0.15)	-0.55 (-1.26, 0.75)	<0.001	<0.001
	Percentage of occupied housing units without a motor vehicle, Median (IQR)	4.93 (2.20, 10.51)	3.57 (1.68, 6.59)	3.70 (1.67, 6.42)	12.15 (5.79, 24.44)	7.55 (4.01, 13.86)	<0.001	<0.001
	Percentage of households without a vehicle, Median (IQR)	0.44 (0.20, 0.71)	0.30 (0.14, 0.56)	0.29 (0.14, 0.52)	0.78 (0.54, 0.91)	0.59 (0.38, 0.79)	<0.001	<0.001
	Percentage of occupied housing units without a telephone, Median (IQR)	1.66 (0.78, 2.91)	1.24 (0.60, 2.23)	1.62 (0.87, 2.62)	2.40 (1.26, 4.10)	1.77 (0.73, 3.39)	<0.001	<0.001
	Percentage of occupied housing units without complete plumbing (log), Median (IQR)	0.00 (0.00, 0.33)	0.00 (0.00, 0.00)	0.00 (0.00, 0.18)	0.00 (0.00, 0.58)	0.00 (0.00, 0.66)	<0.001	<0.001
	Residential history derived - Opportunity Atlas Mean outcome for all children, Median (IQR)	0.54 (0.46, 0.60)	0.59 (0.55, 0.63)	0.54 (0.50, 0.59)	0.43 (0.36, 0.49)	0.46 (0.40, 0.51)	<0.001	<0.001
Social Context	Percentage of persons at least 65 years old, Median (IQR)	0.39 (0.18, 0.64)	0.48 (0.27, 0.69)	0.42 (0.16, 0.67)	0.32 (0.15, 0.54)	0.26 (0.12, 0.46)	<0.001	<0.001
	Percentage of persons 17 years old and younger, Median (IQR)	0.57 (0.30, 0.81)	0.50 (0.26, 0.73)	0.68 (0.36, 0.88)	0.59 (0.30, 0.84)	0.58 (0.29, 0.83)	<0.001	<0.001
	Percentage of civilian noninstitutionalized population older than age 5 with a disability, Median (IQR)	0.33 (0.16, 0.57)	0.22 (0.10, 0.42)	0.37 (0.20, 0.60)	0.56 (0.33, 0.79)	0.29 (0.15, 0.43)	<0.001	<0.001
	Percentage of single-parent households with children less than 18 years old, Median (IQR)	0.48 (0.25, 0.73)	0.32 (0.17, 0.52)	0.44 (0.27, 0.61)	0.79 (0.59, 0.92)	0.69 (0.37, 0.87)	<0.001	<0.001
	Percentage of single, Median (IQR)	14.40 (9.10, 23.29)	10.45 (6.93, 14.84)	12.50 (8.52, 17.62)	28.20 (19.66, 38.58)	21.12 (13.21, 29.47)	<0.001	<0.001
	Single-headed households, Median (IQR)	0.26 (-0.54, 0.88)	0.74 (0.20, 1.10)	0.48 (-0.04, 0.94)	-1.00 (-1.85, -0.30)	-0.19 (-0.70, 0.38)	<0.001	<0.001
	Percentage minority population (i.e., all but white, non-Hispanic), Median (IQR)	0.50 (0.30, 0.76)	0.37 (0.23, 0.53)	0.35 (0.23, 0.49)	0.80 (0.66, 0.91)	0.88 (0.69, 0.95)	<0.001	<0.001
	Percentage of persons at least 5 years old who speak English, "less than well", Median (IQR)	0.52 (0.28, 0.74)	0.45 (0.24, 0.63)	0.37 (0.20, 0.54)	0.69 (0.39, 0.87)	0.91 (0.76, 0.96)	<0.001	<0.001
Crime & Drugs	Residential history derived - Uniform Crime Reports: total adult offenses 1, Median (IQR)	5240.33 (1460.00, 11593.33)	3699.00 (1460.00, 7021.00)	2080.67 (312.67, 5758.67)	5758.67 (1386.33, 7330.33)	71689.34 (20050.00, 71689.34)	<0.001	<0.001
	Residential history derived - Uniform Crime Reports: adult violent crimes 1, Median (IQR)	1084.00 (177.67, 1947.67)	984.00 (283.00, 1709.33)	259.67 (79.00, 1084.00)	1084.00 (227.33, 1947.67)	31135.00 (8847.67, 31135.00)	<0.001	<0.001
	Residential history derived - Uniform Crime Reports: durg abose violations total 1, Median (IQR)	2693.67 (840.67, 6573.67)	2183.33 (840.67, 4589.67)	1214.67 (236.67, 3916.33)	3205.33 (840.67, 6573.67)	50189.00 (18660.00, 50189.00)	<0.001	<0.001
	Residential history derived - Uniform Crime Reports: drug sale total 1, Median (IQR)	493.00 (106.00, 1095.67)	294.00 (106.00, 898.33)	216.67 (45.33, 586.67)	673.00 (102.33, 1235.00)	9937.67 (2695.67, 9937.67)	<0.001	<0.001
	Residential history derived - Uniform Crime Reports: Marijuana sale 1, Median (IQR)	118.00 (43.67, 437.33)	118.00 (60.67, 420.00)	60.00 (12.67, 107.00)	189.33 (58.00, 437.33)	3663.00 (821.00, 3663.00)	<0.001	<0.001

© 2023 American Medical Association. All rights reserved.

Residential history derived - Uniform Crime Reports: drug possession total 1, Median (IQR)	1993.00 (590.33, 5314.67)	1863.67 (590.33, 3935.00)	1035.33 (182.00, 3308.00)	2686.33 (590.33, 4844.67)	40256.67 (15970.33, 40256.67)	<0.001	<0.001
Residential history derived - Uniform Crime Reports: DUI 1, Median (IQR)	2265.00 (751.33, 4499.00)	2125.67 (874.67, 4083.33)	1062.00 (232.33, 2283.00)	2283.00 (631.67, 4499.00)	42953.00 (16475.00, 42953.00)	<0.001	<0.001
Marijuana state law during the same year as the assessment, N (%)							
Recreational law	2659 (25.31)	1648 (40.41)	83 (3.12)	152 (5.73)	776 (69.78)	<0.001	<0.001
Medical law	4902 (46.67)	1773 (43.48)	977 (36.72)	1820 (68.60)	332 (29.86)	<0.001	<0.001
Low THC/CBD (Tetrahydrocannabinol / cannabidiol) law	2853 (27.16)	643 (15.77)	1591 (59.79)	615 (23.18)	4 (0.36)	<0.001	<0.001
No recreational/medical law	90 (0.86)	14 (0.34)	10 (0.38)	66 (2.49)	0 (0.00)	<0.001	<0.001

[a]Comparisons across all 4 SDOH patterns were performed using analysis of variance (ANOVA) test for continuous variables and $\chi 2$ test for categorical variables. Two-tailed *P*-values smaller than 0.05 were considered as the threshold for statistical significance.

© 2023 American Medical Association. All rights reserved.

eTable 3. *P* values of associations between the identified SDOH Patterns and mental health, cognition, and physical health outcomes.

Outcomes		*P* values			
		SDOH Pattern 1	SDOH Pattern 2	SDOH Pattern 3	SDOH Pattern 4
Mental Health Outcome					
Internalizing Problems	Anxious/Depressed	.988	.691	.867	.727
	Withdrawn/Depressed	<.001	.685	<.001	.951
	Somatic Complaints	<.001	.026	.013	.939
	Total Score	.012	.677	.016	.954
Externalizing Problems	Rule-Breaking Behaviors	<.001	.691	<.001	.621
	Aggressive Behaviors	<.001	.763	<.001	.633
	Total Score	<.001	.466	<.001	.629
Problem Behaviors	Social Problems	<.001	.927	<.001	.873
	Thought Problems	.005	.040	.038	.078
	Attention Problems	.027	.086	.211	.434
Mental Health Problem Total Score		<.001	.200	<.001	.992
Suicidal Behaviors	Suicide Attempts	.039	.267	.221	.900
	Suicide Ideation	.842	.420	.894	.481
Cognitive Outcome					
Crystallized Intelligence	Picture Vocabulary	<.001	.002	<.001	.425
	Oral Reading Recognition	<.001	.135	<.001	.405
	Composite Score	<.001	.004	<.001	.339
Fluid Intelligence	Flanker Inhibitory Control	<.001	.015	.039	.523
	List-Sorting Working Memory	<.001	.226	<.001	.005
	Dimensional Change Card Sort	<.001	.059	<.001	.062
	Pattern Comparison Processing Speed	<.001	.695	<.001	.150
	Picture Sequence Memory	.369	.969	.178	.250
	Composite Score	<.001	.061	<.001	.079
Cognitive Intelligence Total Score		<.001	.002	<.001	.090
WISC-V Matrix Reasoning Total Score		<.001	.475	<.001	.005

© 2023 American Medical Association. All rights reserved.

Physical Health Outcome					
General Physical Health	Days exercise ≥60 mins in past 7 days	<.001	.813	<.001	.059
	Body Mass Index (BMI)	<.001	.479	<.001	<.001
Sleep Disorders Scale	Disorders of Initiating and Maintaining Sleep	.012	.055	.065	.085
	Sleep Breathing disorders	<.001	.149	<.001	.743
	Disorder of Arousal	.397	.536	.023	.004
	Sleep-Wake Transition Disorders	.802	.389	.522	.043
	Disorders of Excessive Somnolence	.002	.388	<.001	.071
	Sleep Hyperhidrosis	.012	.286	.061	.827
	Sleep Disorders Scale Total Score	.001	.123	.002	.046

© 2023 American Medical Association. All rights reserved.
