# Supplementary material for: Patterns of Social Determinants of Health and Child Mental Health, Cognition, and Physical Health
Source: JAMA Pediatr. 2023 Oct 16;177(12):1294–305. doi: 10.1001/jamapediatrics.2023.4218 (PMC10580157; doi:10.1001/jamapediatrics.2023.4218)
Supplement: Supplement 2. — Data Sharing Statement [file jamapediatr-e234218-s002.pdf]

Data Sharing Statement

Xiao. Patterns of Social Determinants of Health and Child Mental Health, Cognition, and Physical Health. *JAMA Pediatr*. Published October 16, 2023.
doi:10.1001/jamapediatrics.2023.4218

Data
Data available: No
